# Novel Role for p110β PI 3-Kinase in Male Fertility through Regulation of Androgen Receptor Activity in Sertoli Cells

**DOI:** 10.1371/journal.pgen.1005304

**Published:** 2015-07-01

**Authors:** Julie Guillermet-Guibert, Lee B. Smith, Guillaume Halet, Maria A. Whitehead, Wayne Pearce, Diane Rebourcet, Kelly León, Pascale Crépieux, Gemma Nock, Maria Strömstedt, Malin Enerback, Claude Chelala, Mariona Graupera, John Carroll, Sabina Cosulich, Philippa T. K. Saunders, Ilpo Huhtaniemi, Bart Vanhaesebroeck

**Affiliations:** 1 UCL Cancer Institute, University College London, London, United Kingdom; 2 Centre de Recherche en Cancérologie de Toulouse UMR1037, INSERM, BP84225, Toulouse, France; 3 Université Toulouse III-Paul Sabatier, Toulouse, France; 4 MRC Centre for Reproductive Health, University of Edinburgh, The Queen’s Medical Research Institute, Edinburgh, United Kingdom; 5 CNRS, UMR 6290, Institut de Génétique et Développement de Rennes, Rennes, France; 6 Université Rennes 1, UEB, SFR BIOSIT UMS 3480, Faculté de Médecine, Rennes, France; 7 Physiologie de la Reproduction et des Comportements, UMR 7247 INRA—CNRS—Université de Tours, Nouzilly, France; 8 Astra Zeneca, Research and Development, Mölndal, Mölndal, Sweden; 9 Centre for Molecular Oncology, Barts Cancer Institute, Queen Mary University of London, Charterhouse Square, London, United Kingdom; 10 Vascular Signalling Laboratory, Institut d´Investigació Biomèdica de Bellvitge (IDIBELL), Gran Via de l’Hospitalet 199–203, 08908 L´Hospitalet de Llobregat, Barcelona, Spain; 11 Department of Anatomy and Developmental Biology, Monash University, Melbourne, Australia; 12 Astrazeneca Oncology iMED, Alderley Park, Macclesfield, Cheshire, United Kingdom; 13 MRC Centre for Inflammation Research, University of Edinburgh, The Queen’s Medical Research Institute, Edinburgh, United Kingdom; 14 Institute of Reproductive and Developmental Biology, Imperial College London, Hammersmith Campus, London, United Kingdom, and Department of Physiology, University of Turku, Turku, Finland; University of Torino, ITALY

## Abstract

The organismal roles of the ubiquitously expressed class I PI3K isoform p110β remain largely unknown. Using a new kinase-dead knockin mouse model that mimics constitutive pharmacological inactivation of p110β, we document that full inactivation of p110β leads to embryonic lethality in a substantial fraction of mice. Interestingly, the homozygous p110β kinase-dead mice that survive into adulthood (maximum ~26% on a mixed genetic background) have no apparent phenotypes, other than subfertility in females and complete infertility in males. Systemic inhibition of p110β results in a highly specific blockade in the maturation of spermatogonia to spermatocytes. p110β was previously suggested to signal downstream of the c-kit tyrosine kinase receptor in germ cells to regulate their proliferation and survival. We now report that p110β also plays a germ cell-extrinsic role in the Sertoli cells (SCs) that support the developing sperm, with p110β inactivation dampening expression of the SC-specific Androgen Receptor (AR) target gene *Rhox5*, a homeobox gene critical for spermatogenesis. All extragonadal androgen-dependent functions remain unaffected by global p110β inactivation. In line with a crucial role for p110β in SCs, selective inactivation of p110β in these cells results in male infertility. Our study is the first documentation of the involvement of a signalling enzyme, PI3K, in the regulation of AR activity during spermatogenesis. This developmental pathway may become active in prostate cancer where p110β and AR have previously been reported to functionally interact.

## Author Summary

Class I PI3Ks are important signalling enzymes and drug targets in cancer and inflammation. We report that p110α and p110β, the two ubiquitously expressed class I PI3K isoforms, control fertility, with no evidence for such a role for p110δ, a PI3K highly expressed in leukocytes. Infertility is therefore a possible but reversible side-effect of PI3K-targeted therapies. Using a new mouse model of systemic p110β inactivation, we found that p110β is critical for ensuring the quality of eggs in females and for sperm formation in males. p110β inactivation leads to a specific blockade in sperm development, without affecting the spermatogenic stem cell pool. This, together with the observation that p110β inactivation has no detectable organismal side effects in the adult stage, makes this kinase a potential drug target for a male contraceptive. Besides its previously reported role in the spermatogenic cells themselves, we now report that p110β also regulates the action of androgens, the male sex hormones, specifically in the Sertoli cells that surround the developing sperm, without affecting androgen action in other tissues. In cancer, however, p110β may acquire the capacity to regulate androgen action in tissues other than Sertoli cells, as was previously documented in prostate cancer.

## Introduction

Upon stimulation of cells with extracellular ligands, class I phosphoinositide 3-kinases (PI3Ks) generate lipids that modulate the function of a range of signalling proteins, including protein kinases (such as Akt/PKB), regulators of small GTPases and adaptor proteins. These PI3K effectors regulate an array of cellular outputs, including cell cycle progression, cell survival, metabolism, translation, transcription and cell motility. Class I PI3Ks have been implicated in cancer, immunity and metabolism, and are the subject of active drug development efforts [1–4].

Mammals have four class I PI3K catalytic isoforms (called p110s) that occur in a heterodimeric complex with a regulatory subunit. Class IA catalytic subunits (p110α, β and δ) are bound to an SH2 domain-containing p85 regulatory subunit, that binds to Tyr phosphorylated membrane-associated proteins, whereas the p84 and p101 regulatory subunits lack SH2 domains and link the single class IB PI3K, p110γ, to G protein-coupled receptors (GPCRs). Tyrosine kinases activate p110α, β and δ, whereas GPCRs regulate p110β and γ. While p110α and β are ubiquitously expressed, p110γ and δ are mainly found in leukocytes but can also be expressed at lower levels in other cell types [5].

Studies using PI3K mutant mice and pharmacological PI3K inhibitors have largely focused on p110α, γ and δ and revealed isoform-selective signalling functions for the class I PI3Ks [1,6,7]. Comparatively less is known about p110β. Several genetic mouse models of p110β inactivation have been created, including mice with full [8] or partial [9,10] deletion of the p110β gene, and mice that produce a hybrid mouse/human inactive p110β protein [11,12]. Conflicting data have been obtained using these different mouse models: whereas one p110β gene deletion model [8] displays a fully penetrant, very early embryonic lethality (at embryonic day E3.5), p110β gene deletion using another strategy [9,10], or its replacement with a cDNA encoding for an inactive p110β enzyme [11,12], show only partial embryonic lethality that occurs at later stages of development. Mice that survived full p110β inactivation were apparently normal [11] but showed a mild growth retardation that normalized after 6 months of age, at which stage these mice became mildly insulin-resistant, with increased blood glucose levels [11]. Homozygous inactivation of p110β with this strategy had no impact on female fertility but led to male sterility [12].

The PI3K signalling pathway has previously been implicated in germ cell-intrinsic regulation of fertility. This was documented through conditional inactivation of PTEN in the female and male germlines [13–15] or by inactivation of PDK1 in the male germline [15]. Organismal inactivation of Akt1 or Akt2 has also been reported to lead to reduced testis size, reduced male fertility and increased apoptosis in male germ cells [16–18]. Mice in which the endogenous c-kit tyrosine kinase receptor no longer binds class IA PI3Ks further revealed a role for this group of PI3Ks in male [19,20] and female fertility [19]. Subsequent studies using mice with systemic inactivation of p110β [11] suggested that this kinase provides PI3K activity downstream of c-kit in male germ cells [12], although a germ-cell-intrinsic role of p110β remains to be formally proven.

In this study, we have investigated the organismal role of p110β by inactivating it in mice using a gene targeting strategy that we previously applied to p110α and p110δ [21–24]. We have created a knockin mouse line in which a point mutation in the kinase domain renders the endogenous p110β inactive but preserves its expression levels, thus mimicking the action of a kinase inhibitor. Such systemic inactivation of p110β in mice resulted in a substantial, although not fully penetrant, embryonic lethality, with the surviving mice showing defects in fertility, especially in males. In addition to the previously suggested role for p110β in the germ cell compartment [12], we find that p110β also has a germ cell-extrinsic role in the regulation of fertility, namely by regulating androgen receptor (AR) gene expression in SCs, which is known to be critical for the proper development of the male germ cells. We also present evidence of a role for p110α, the other ubiquitously expressed class I PI3K, in male and female fertility.

## Results

### Generation of a mouse line with inactive p110β

Using a strategy previously applied to p110α and p110δ [22,24], we created a mouse line in which endogenous p110β is converted to a kinase-dead protein. This was achieved by introducing a germline point mutation in *Pik3cb*, the gene encoding p110β, which converts the critical ATP-binding DFG motif in the p110β kinase domain to AFG (**S1 Fig** and **S1 Text**). Experiments using mice homozygous for this mutant p110β (referred to as p110β^D931A/D931A^ mice) showed that the p110β^D931A^ protein lacked catalytic activity (**S2A Fig**) but was expressed at the same level as wild-type (WT) p110β (**S2B Fig**). In addition, it did not affect expression of p85 (**S2B Fig**) nor the expression (**S2B Fig**) or activity (**S2A Fig**) of p110α. In heterozygous p110β^D931A/WT^ mice, p110β lipid kinase activity was reduced by approximately 50%, with the remaining activity being significantly sensitive to the p110β-selective inhibitor TGX-221 (**S2C Fig**). Loss of p110β activity did not decrease total PI3K activity present in phosphoTyr-peptide precipitates (which pull down all p85 subunits; **S2A Fig**) nor the basal phosphorylation of Akt/PKB on S473 in the lungs and testes (**S2B Fig**). Taken together, these data show that germline conversion of p110β to the p110β^D931A^ form inactivates this kinase without affecting the expression or activity of other, non-targeted, PI3K isoforms.

### p110β inactivation leads to substantial loss of embryonic viability

Intercrosses of p110β^D931A/WT^ mice yielded a significantly lower than expected fraction of homozygous p110β^D931A/D931A^ mice born, based on a normal Mendelian distribution, both on a mixed C57BL/6 x 129S2/Sv or on a pure C57BL/6 background (10% and 1% *versus* 25% expected, respectively) (**S3A Fig**). The reason for the lethality of p110β^D931A/D931A^ embryos is unknown at the moment. Indeed, it was not possible to identify a specific time point of embryonic lethality, as embryos were found to die at different stages of embryonic development (**S3A Fig**). This is in stark contrast to the fully penetrant embryonic lethality of homozygous p110α kinase-dead mice that all die at E10.5 [22].

### Homozygous p110β kinase-dead males are infertile

**Fig 1:**
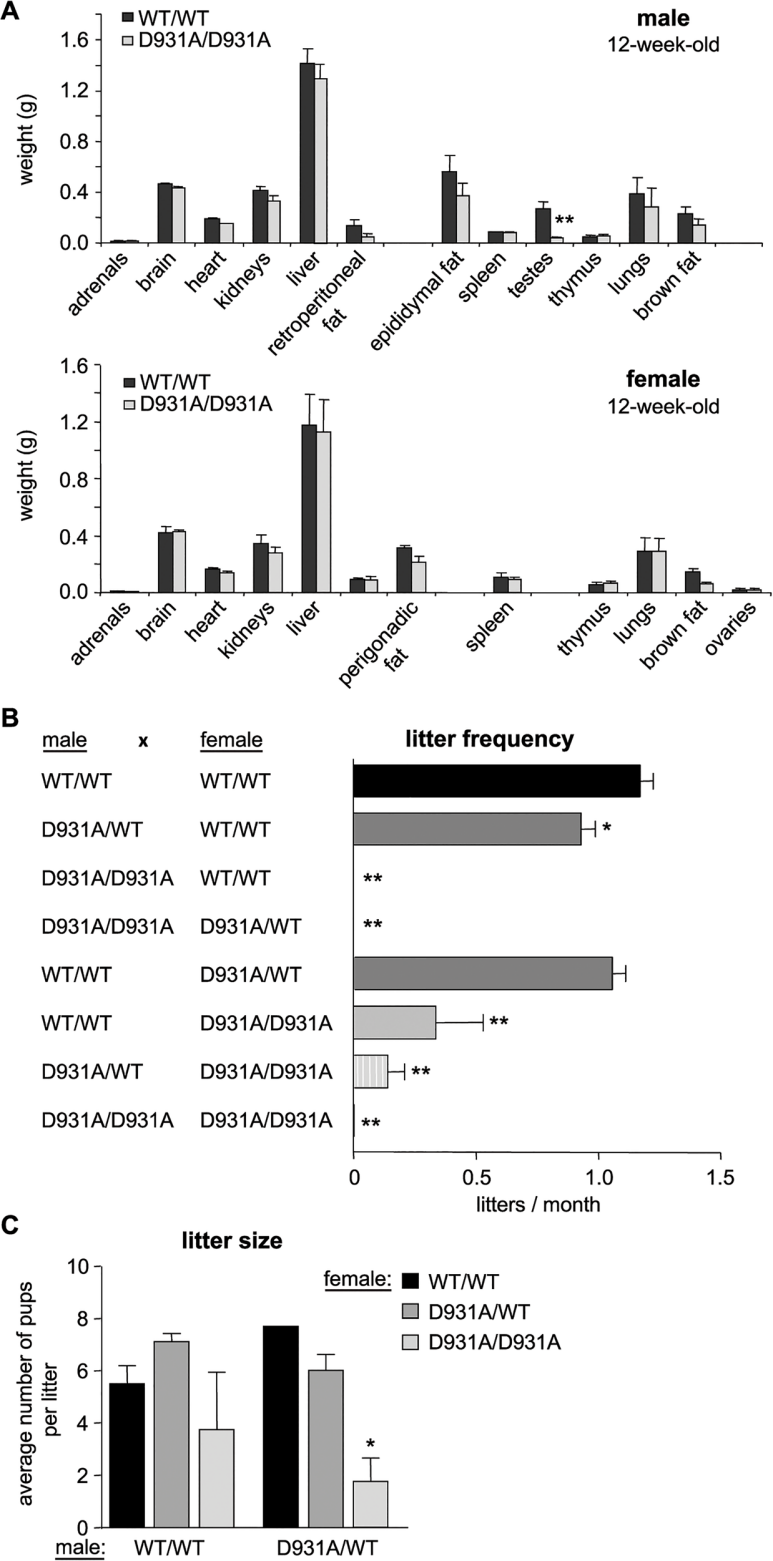
p110β kinase activity positively regulates female and male fertility. **A**) Weight of organs in 12-week-old mice (n = 4). **B**) Mice with the indicated genotype were bred for a 6-month period (cages of 2 females with 1 male; > 3 couples) and the average number of litters per month was assessed. Mann-Whitney: **, p<0.01. **C**) Average size of litters obtained from breeding pairs (2 females with one male for ≥4 months). Unpaired t-test: *, p<0.05; **, p<0.01.

p110β^D931A/D931A^ embryos (**S3B Fig**) and 4-week-old male mice (**S3C Fig**) showed a mild growth delay. However, no weight differences were seen in male or female adult mice (**S3D Fig**). Necropsy and comprehensive histological analysis (see **S1 Table** for a list of organs analyzed) of ~6-month-old p110β^D931A/D931A^ mice did not reveal any detectable alterations or pathology, apart from reduced size (**S4 Fig**) and altered histology (see below) of the testes (**Fig 1A** shows the organ weights of 12-week-old mice). p110β^D931A/D931A^ males, on both pure and mixed genetic backgrounds, were found to be sterile upon mating with WT females (**Fig 1B**), suggesting oligo- or azoospermia. p110β^D931A/WT^ males, when mated with WT females, also showed a 20% reduction in litter frequency compared to WT males (**Fig 1B**), although the litter size was unaltered (**Fig 1C**).

### Maternal p110β activity contributes to effective transitioning of the 2-cell embryo to the morula/blastocyst stage

**Fig 2:**
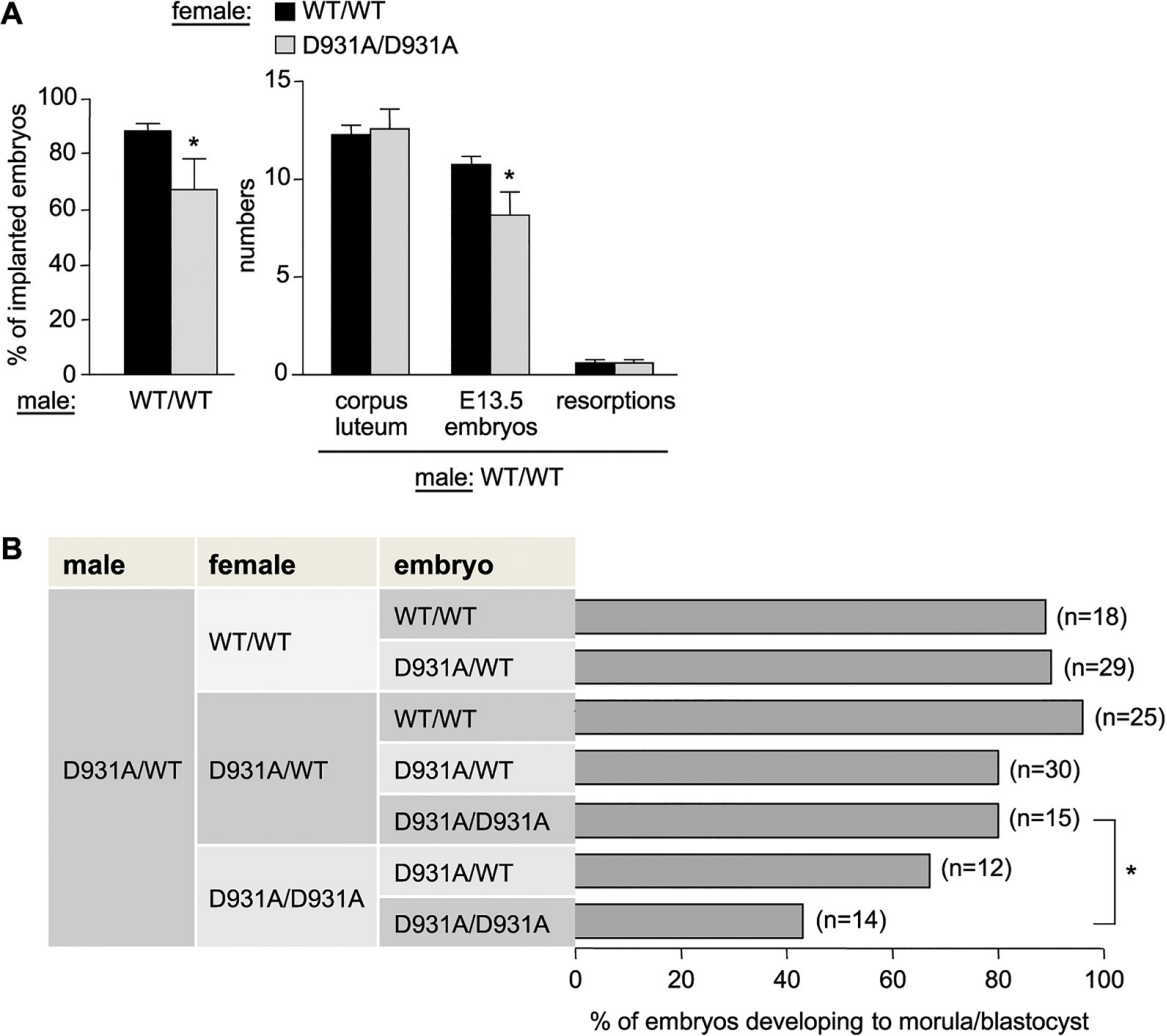
Maternal and embryonic p110β kinase activity regulate preimplantation embryogenesis. **A**) Females of the indicated genotype were crossed with WT males (n = 5 females crossed with 2 different males). The percentage of ovulations which became implanted embryos (left panel) was calculated as follows: [numbers of implanted E13.5 embryos + number of resorptions]/corpus luteum numbers in the ovaries (indicative of the number of ovulated oocytes)] x 100 (right panel). Mann-Whitney: *, p<0.05. **B**) Females of the indicated genotype were superovulated and mated with a p110β^**D931A/WT**^ male. Two-cell embryos were recovered from the oviducts and cultured *in vitro* for 4 days, at which time embryos were scored for development to the morula/blastocyst stage or any earlier developmental stage, and genotyped. Mann-Whitney: *, p<0.05.

Female p110β^D931A/D931A^ mice also showed a substantial reduction in fertility. Indeed, p110β^D931A/D931A^ females, when crossed with WT males, had a reduction of 70% in their capacity to have recurrent litters (0.34 litters born per month *versus* 1.20 in intercrosses of WT mice; **Fig 1B**), a reduced litter size when crossed with p110β^D931A/WT^ males (**Fig 1C**) and a 24%-reduction in the percentage and absolute number of ovulated oocytes that made it to E13.5 embryos (**Fig 2A**).

p110β^D931A/D931A^ females showed normal follicle maturation (**S5A Fig**) and oestrus cycles (**S5B Fig**) and generated the same number of 2-cell embryos upon superovulation and mating with WT males (**S5C Fig**), suggesting normal ovulation in these mice. However, 2-cell p110β^D931A/D931A^ embryos recovered from p110β^D931A/D931A^ females had a decreased *in vitro* ability to develop into morula and blastocysts and to survive *ex vivo*, compared to p110β^D931A/D931A^ embryos generated by p110β^D931A/WT^ females (**Fig 2B**; representative *ex vivo* cultures and genotyping results are shown in **S5D and S5E Fig**). Taken together, these data indicate that the lack of embryonic p110β activity is not, *per se*, detrimental to preimplantation development to blastocyst if the female is heterozygous for p110β inactivation. Thus, a maternal pool of p110β, provided by the oocyte cytoplasm and/or the host environment, likely participates in healthy embryonic development to blastocyst.

### p110β activity is essential for the first round of spermatogenic meiosis after birth

**Fig 3:**
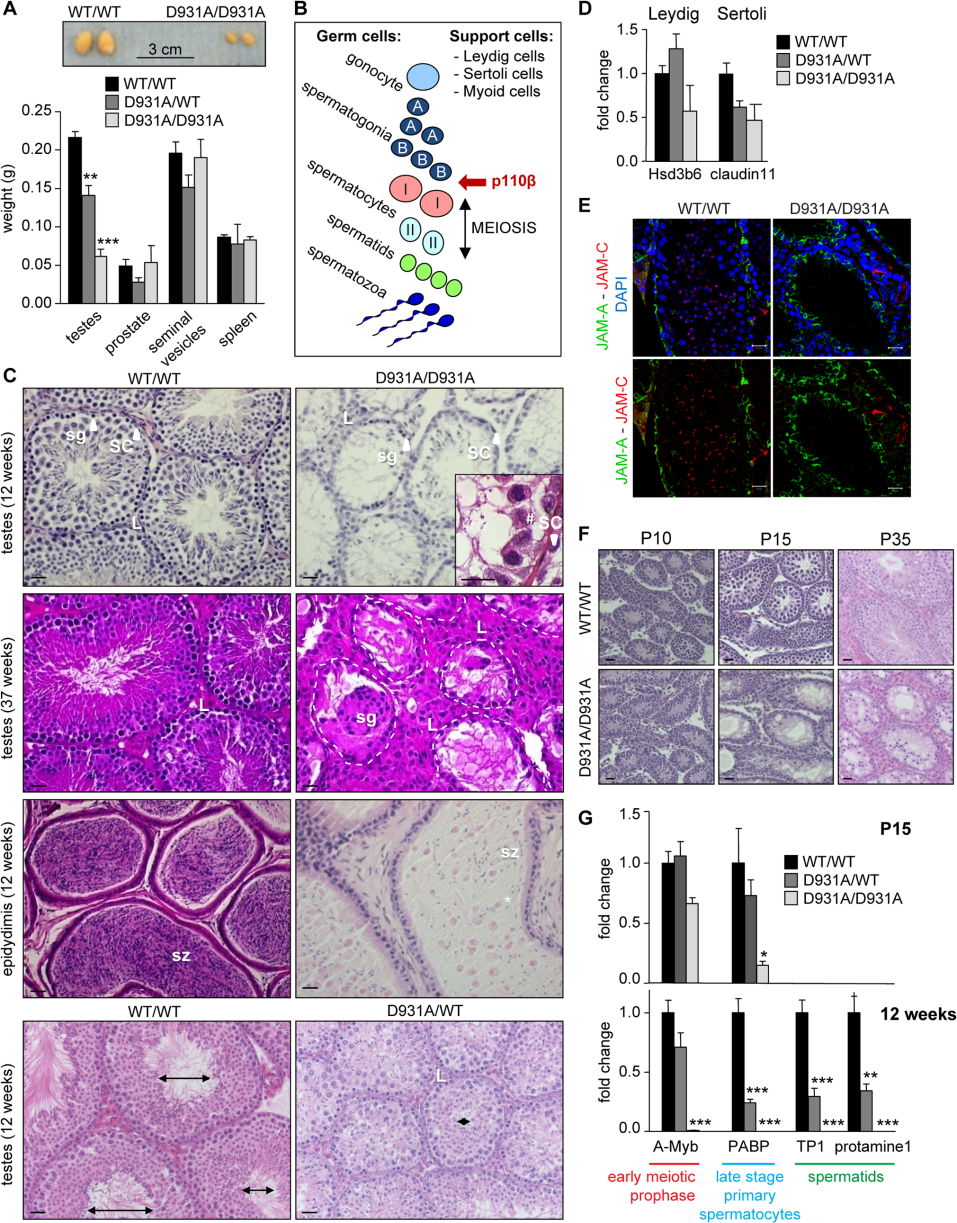
Systemic organismal inactivation of p110β reveals a role for p110β in SCs. **A**) Weight of reproductive organs in 12-week-old mice (n>6). Mann-Whitney: **, p<0.01; ***, p<0.001. **B**) Schematic of the spermatogenic lineage, testis support cells and the earliest stage of interference by p110β. **C**) H&E-stained sections of 12-week-old and 37-week-old testis and 12-week-old epididymis. sg, spermatogonia; sz, spermatozoa; SC, Sertoli cell; L, Leydig cell; #, presence of germ cells in p110β^**D931A/D931A**^ seminiferous tubules; *, abnormally detached undifferentiated spermatids. Lumen size is indicated by double ended arrows. Scale: 20 μm. **D**) mRNA expression levels of the indicated genes at 12-week-old as determined by RT-qPCR normalized with 18S expression and corrected for total testis weight (n = 4). Student's t-test: non-significant. **E**) Immunofluorescence staining for JAM-A (Sertoli cell-specific protein localized in tight junctions) and JAM-C (spermatid-specific protein) counterstained with DAPI on cryosections of testes of 12-week-old mice (n = 3) [64]. * indicate cell membranes of Sertoli cells; white arrow are JAM-C positive spermatids. Scale: 20 μm. **F**) H&E-stained sections of testes during post-natal development (P10: n = 4; P15: n = 2; P35: n = 3). Scale: 20 μm. **G**) mRNA expression levels of the indicated genes at P15 (top panel) and 12 weeks (bottom panel) as determined by RT-qPCR normalized with 18S expression and corrected for total testis weight (n = 4). Student's t-test: *, p<0.05; **, p<0.01; ***, p<0.001.

We next analyzed the p110β^D931A/D931A^ male sterility phenotype in more detail. At 12 weeks of age, the testes were the only organs reduced in weight (by more than 70% compared to WT testes) in the p110β^D931A/D931A^ males (**Figs 1A** and **3A**). Testes of p110β^D931A/D931A^ males had descended normally into the scrotum (**S6 Fig**), indicating that the first intrauterine/early postnatal peaks of testosterone production had led to correct perinatal testicular development [25]. Serum levels of testosterone and luteinizing hormone (LH) were not significantly altered (**S7 Fig**), in accordance with the unaffected organ weight of other androgen target tissues, including prostate, seminal vesicles and epididymal and retroperitoneal fat (**Figs 1A** and **3A**). However, compared to WT controls, p110β^D931A/D931A^ males had a 37% increase in the serum levels of follicle-stimulating hormone (FSH; **S7 Fig**), a phenomenon known to occur upon spermatogenic arrest [25].

The appearance and size of the interstitial tissue, that surrounds the germ cell-containing seminiferous tubules (**Fig 3B**) and is composed of the testosterone-producing Leydig cells (labelled L in **Fig 3C** and **S8 Fig**), were analysed in WT and p110β^D931A/D931A^ males. p110β inactivation did not lead to significant changes in the mRNA expression levels of the Leydig cell marker Hsd3b6 (3-β-hydroxysteroid dehydrogenase 6), the localisation of the HSD3B protein (**Fig 3D** and **S8 Fig**) or the number of HSD3B-positive cells (**S8E Fig**). However, a tendency for an increase in Leydig cell numbers was observed in aged p110β^D931A/D931A^ mice.

Homozygous inactivation of p110β did also not affect the presence of SCs in the testes (**Fig 3C**). In addition, no differences were seen in the expression of the SC-specific mRNA claudin 11 in 12-week-old mice (**Fig 3D**), or in the number of SC-marker SOX9-positive cells during testes development and aging (**S8E Fig**). In addition, the localization of the adhesion molecule JAM-A was not affected in p110β^D931A/D931A^ SCs (**Fig 3E**), indicative of an intact blood-testis barrier of the seminiferous tubules.

During spermatogenesis, specialized stem cells, called spermatogonia, undergo meiosis and cell differentiation into, sequentially, spermatocytes, spermatids and spermatozoa (**Fig 3B**) [26]. Spermatogonia reside on the basement membrane of the seminiferous tubules and are surrounded by supporting SCs. To understand the spermatogenic defect caused by p110β inactivation in more detail, we analysed the differentiation of spermatogonia. The first round of meiosis occurs between post-natal stage P10 to P15. The spermatogenic cell pool was altered in p110β^D931A/D931A^ males at P10, the known time point of onset of meiosis, as evidenced by staining of the germ cell marker DDX4 (**S8A Fig**). At P15, the H&E staining and DDX4 staining of p110β^D931A/D931A^ testes revealed a clear depletion of germ cells, coinciding with the known time point of primary spermatocyte appearance, and further observed at P35 (**Fig 3F** and **S8B Fig**). At 12 weeks of age, the seminiferous tubules of p110β^D931A/D931A^ testes contained only a few spermatogenic cells, many of which were detached (**Fig 3C** and **S8 Fig**, DDX4 marker), and no JAM-C positive cells (**Fig 3E**), indicative of a massive loss of spermatids. However, a low concentration of isolated round and elongating spermatids and few mature spermatozoa were present in the cauda epididymis of testes of 12-week-old p110β^D931A/D931A^ mice (**Fig 3C**). At 37 weeks of life, most of the tubular cross-sections of p110β^D931A/D931A^ testes displayed a ‘SC only’ appearance (**Fig 3C**).

We next analysed the gene expression of selected differentiation markers (A-Myb, PABP, TP1 and protamine1) in the testes. At P15, the expression of A-Myb, a marker of the early meiotic prophase, was not significantly altered in p110β^D931A/D931A^ testes (**Fig 3G**) whereas expression of PABP, a marker for late stage spermatocytes, was severely reduced (**Fig 3G**), suggesting an altered progression in germ cell differentiation. The expression of all these markers was undetectable at 12 weeks of age (**Fig 3G**).

In heterozygous p110β^D931A/WT^ males, the expression of both A-Myb and PABP was similar to that in WT mice at P15 (**Fig 3G**). In adult heterozygous p110β^D931A/WT^ males that have a modestly reduced fertility (**Fig 1B**) and a 35% reduction in testis size compared to WT mice (**Fig 3A**), the diameter and lumen size of the seminiferous tubules were severely reduced at 12 weeks of age (**Fig 3C, bottom panel**). Accordingly, the expression of markers of late primary spermatocyte (PABP) and spermatid (TP1 and protamine1) stages was strongly reduced in these mice, while the expression of A-Myb was unaffected (**Fig 3G**), indicative of unaffected spermatogenesis in young p110β^D931A/WT^ males but a progressive depletion of spermatogenic germ cells upon aging.

In summary, our data reveal an essential role for p110β in early meiosis (**Fig 3B**). The presence of small numbers of post-meiotic germ cells in the epididymis of 12-week-old p110β^D931A/D931A^ males suggests that meiosis is possible in the absence of p110β activity but that it occurs with a possible altered efficiency, loss-of-contact and sloughing off of the germinal lineage from SCs before timely spermiogenesis, resulting in severely impaired sperm production.

### Selective inactivation of p110β in SCs leads to male sterility but does not affect germ cell composition

The detachment of the germinal cell lineage and inefficient primary spermatocyte formation, observed in p110β^D931A/D931A^ testes at P15, are both phenomena that are known to be controlled by SCs [25,27]. This suggests that p110β activity could be important in SCs, despite the lack of obvious histological differences in p110β^D931A/D931A^ SCs (**Fig 3C**) and the unaltered staining for the SC-specific marker SOX9 (**S8 Fig**).

**Fig 4:**
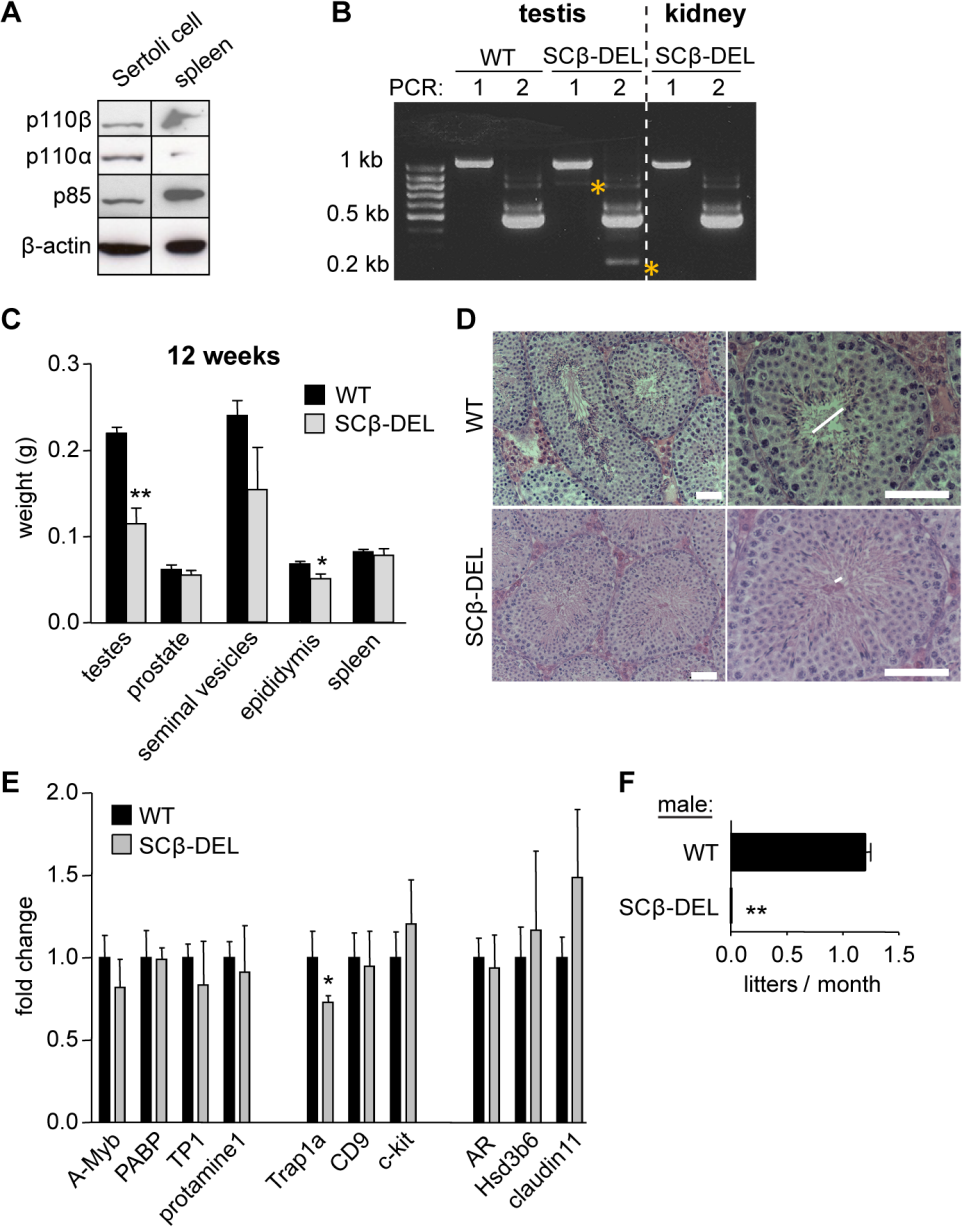
Tissue-specific inactivation of p110β in SCs causes male sterility. **A**) p110 protein expression in primary rat SC culture. **B**) Following genotyping, AMH-Cre^**+**^ testes were checked for *Pik3cb* recombination levels by a RT-nested PCR. No recombination was detected in Cre^**-**^ testis (not shown) or in other organs. PCR 1 for *Pik3cb* exons 16–24: WT 1067 bp; β-DEL 797 bp; PCR 2 for *Pik3cb* exons 19–23: WT 474 bp; β-DEL 204 bp. β-DEL amplicons in both PCRs are indicated with a yellow star, and were only detected after the second PCR, consistent with the fact that SCs are less abundant in testis than the spermatogenic lineage. **C**) Weight of the indicated organs in recombined AMH-Cre^**+**^p110β^**flox/flox**^ mice, referred to as SCβ-DEL (n = 5). Mann-Whitney: *<p0.05, **, p<0.01. **D**) Histology of 12-week-old SCβ-DEL testes (n = 3). Lumen size is indicated by horizontal lines. Scale 50 μm. **E**) mRNA expression levels of the indicated genes in testes of 12-week-old mice (n = 4) mice as determined by RT-qPCR normalized with 18S expression and corrected for total testis weight (n = 4). Student's t-test: *, p<0.05. **F**) Breeding efficiency of WT and SCβ-DEL males crossed with WT C57BL/6 females (n = 5). Despite some variability in the histological phenotype, all mice with a recombined *Pik3cb* were found to be sterile. Unpaired t-test: **, p<0.01.

To assess the possible role of p110β expressed in SCs (**Fig 4A**), we crossed mice with a conditional inactivating allele of p110β (p110β^flox^ [9]) with mice expressing the Cre recombinase under the control of the SC-specific AMH promoter (AMH-Cre mice [28]). 12-week-old mice with recombined *Pik3cb* loci in AMH-Cre-expressing SCs (referred to as SCβ-DEL; **Fig 4B**) had a decrease in the weight of the testes (48%) and epididymis (25%) with no alterations in the weight of the prostate, seminal vesicles or spleen (**Fig 4C**). The diameter of the seminiferous tubules was also reduced in SCβ-DEL testes (**Fig 4D**), but, in contrast to p110β^D931A/D931A^, the germ cell composition of the testes was unaltered (**Fig 4D**). In line with this, the expression of markers of each germ cell stage was unchanged in SCβ-DEL testes at 12 weeks of age, with the exception of the primordial germ cell marker Trap1a (**Fig 4E**). Importantly, however, none of the SCβ-DEL mice gave rise to offspring when crossed with WT females (**Fig 4F**). These data show that, like systemic organismal inactivation, SC-specific inactivation of p110β leads to male sterility but likely through a different mechanism than primary spermatocyte formation. These data suggest that, in addition to its key role in SCs, p110β may also regulate spermatogenesis in a germ cell-intrinsic manner.

### The male fertility phenotype upon p110β inactivation shares similarities with SC-specific deletion of the androgen receptor (AR)

We next searched for the mechanism by which p110β activity regulates SC function. The AR has a key role in controlling spermatogenesis. As germ cells do not express the AR, androgen regulates fertility indirectly through regulating gene expression in SCs, which influences germ cell maturation. Testosterone binding to the AR in SCs is a key signal in the regulation of the first round of meiosis in male gametogenesis during early postnatal development [25,29]. Other roles of testosterone in SCs include the attachment of developing spermatids to the SCs [30,31] and lumen formation of seminiferous tubules [32], both of which were found to be affected by full inactivation of p110β. Indeed, the phenotype of p110β^D931A/D931A^ testes was reminiscent of some aspects of SC-specific AR knockout (SCARKO) mice [30]. Compared to p110β^D931A/D931A^ mice, in SCARKO mice the reduction in the diameter of the seminiferous tubules is milder, and sperm cell differentiation is blocked at a later (round spermatid) stage (**Fig 3B**). SCs start to express the AR at P4 [33], with increased expression at P15 [32], coinciding with the stage at which the p110β^D931A/D931A^ testis phenotype becomes apparent (**Fig 3F and 3G** and **S8 Fig**). We therefore hypothesized that p110β that is expressed in SCs could regulate some aspects of AR activity.

**Fig 5:**
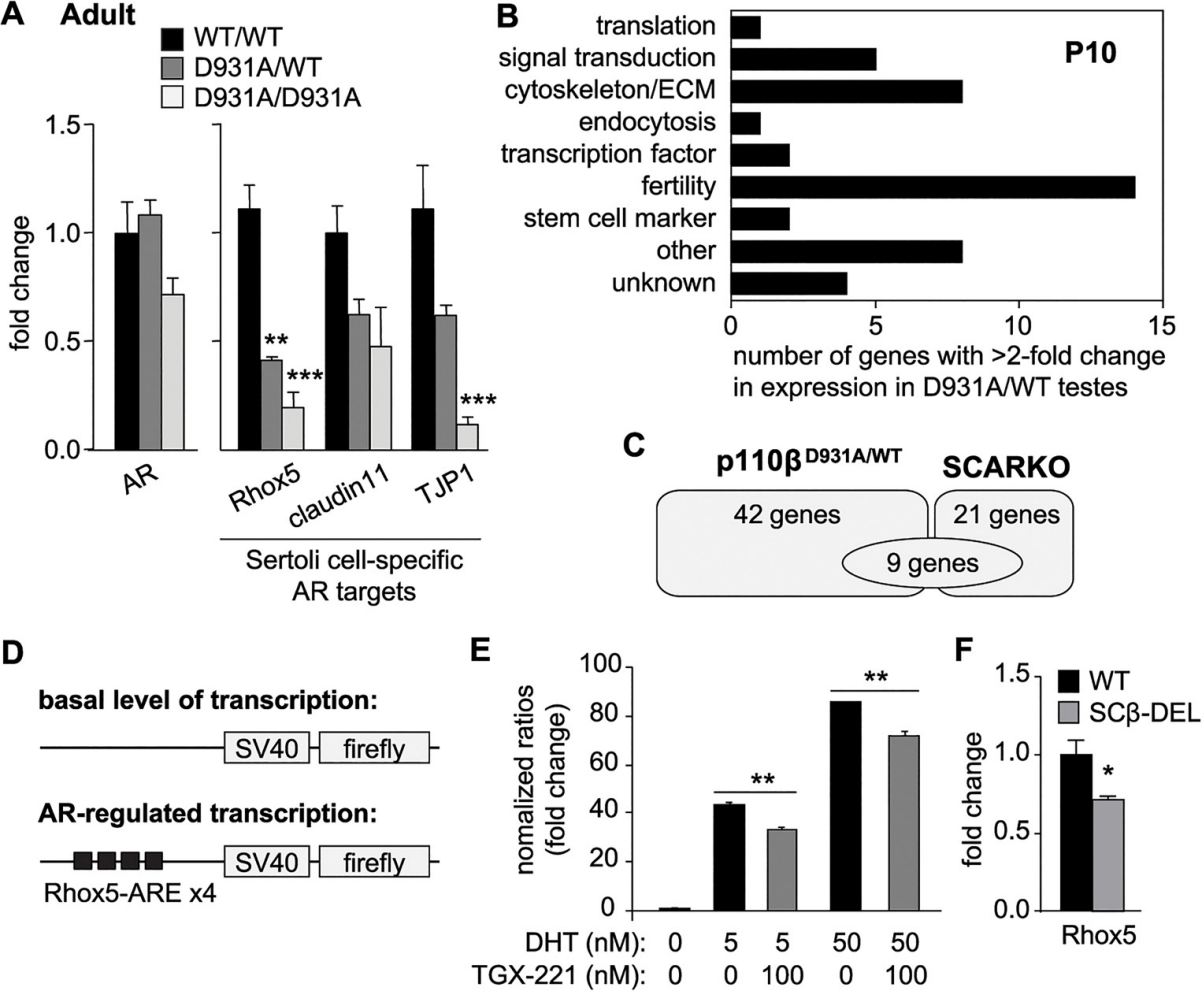
p110β activity regulates AR mRNA targets specifically in SCs. **A**) mRNA expression of selected markers in adult testes of mice with the indicated genotypes as determined by RT-qPCR and normalized with 18S expression and corrected for total testis weight (n>4). Student's t-test: **, p<0.01; ***, p<0.001. **B**) Functional classification of genes with >2-fold altered expression in testes of p110β^**D931A/WT**^ males, compared to WT mice (for details see **S3 Table**). **C**) Gene expression pattern in p110β^**D931A/WT**^ testis compared to SCARKO testis [38] (for details see **S4 Table**). **D**) Schematic representation of plasmids transiently transfected in MSC-1 cells. **E**) Transient co-transfection of Rhox5-AREs luciferase reporter and Flag-AR of MSC-1 cells. 5α-DHT (5 and 50 nM) was used to activate AR transactivation activity. TGX-221 was used at 100 nM. Results are presented as induction factors (averages ± SEM of ≥3 independent experiments performed in triplicate). Student's t-test: **, p<0.01. **F**) mRNA expression of Rhox5 in testes of mice of the indicated genotypes (n>4) as determined by RT-qPCR and normalized with 18S expression and corrected for total testis weight. Student's t-test: *, p<0.05.

Systemic inactivation of p110β in mice did not affect AR mRNA expression in the testes (**Fig 5A, left panel**). In contrast, the mRNA expression of the SC-specific AR-responsive homeobox-gene *Rhox5* was reduced in both p110β^D931A/D931A^ and p110β^D931A/WT^ 12-week-old testes (**Fig 5A, right panel**). The expression of Rhox5 is critical for the full efficiency of meiosis [29,32,34]. p110β inactivation also led to reduced expression of other SC-specific AR targets in adult testes, including TJP1 (Tight Junction Protein 1) and claudin11 (a transmembrane protein important for tight junctions), although the reduction in the expression of claudin11 did not reach statistical significance (**Fig 5A, right panel**). In contrast, systemic p110β inactivation did not affect the expression of Leydig cell-specific AR target genes such as *Hsd3b6* [35] (**Fig 3D**), in line with the lack of obvious defects in this cell population upon p110β inactivation (**Fig 3C and 3D** and **S8 Fig**).

The AR also has a pivotal role in the regulation of extragonadal reproductive glands, muscle mass, fat deposition and bone or brain function [36,37], none of which were notably affected in p110β^D931A/D931A^ mice (**Fig 3A**). Taken together, these data indicate that p110β regulates a subset of AR target genes, specifically in SCs.

### Gene network analysis in testes identifies AR-regulated genes in SCs as p110β targets

In order to gain further insight into the functional link between p110β and AR, we performed an unbiased global gene expression analysis in WT and p110β^D931A/WT^ testes at P10. This early time point was selected in order to investigate the events associated with the initiation of the p110β-associated fertility phenotype. The use of p110β^D931A/WT^ testes is also expected to reveal the primary transcriptional targets of AR regulated by this PI3K, as homozygous inactivation of p110β likely results in ‘knock-on’ effects on spermatogenesis regulation. One such effect is the induction of the FSH/LH feedback loop that arises as a consequence of impaired production of spermatozoa. Indeed, the plasma levels of FSH were significantly increased in p110β^D931A/D931A^ but not p110β^D931A/WT^ males (**S7 Fig**). However, a drawback of using p110β^D931A/WT^ mice is the potentially low magnitude of change in the gene expression as compared to WT mice. For this reason, we considered a 2-fold difference in gene expression significant in this setting.

The expression of 42 genes was found to be altered ≥2-fold between WT and p110β^D931A/WT^ testes (17 genes downregulated, p-value 0.0052–0.00013; 25 upregulated, p-value 0.015–0.00013; **S2 Table**). The functions of these genes span various biological contexts, with genes known to regulate fertility forming the main group (**Fig 5B** and **S3 Table**). A comparison of the gene expression profiles of p110β^D931A/WT^ and SCARKO P10 testes [38] **(Fig 5C** and **S4 Table**) showed that, of the 21 genes significantly modified in SCARKO, 9 also showed an altered expression between WT and p110β^D931A/WT^ testes. p110β activity thus regulates the expression of a fraction of known SC-specific AR-regulated genes, while other genes regulated by p110β appear not to be dependent on Sertoli-cell specific AR activity. This is indicative of the AR in SCs having p110β-independent functions but also of p110β having 1) AR-independent functions in SCs and 2) SC-independent functions in the testes, such as the regulation of germ cell survival and proliferation.

### p110β activity modulates AR-regulated gene transcription

The AR resides in the cytoplasm and upon binding to testosterone translocates to the nucleus where it binds to its DNA-response elements in the promoter or enhancer regions of androgen target genes. To demonstrate that p110β activity has the ability to regulate the genomic functions of AR, we transiently transfected the mouse SC line MSC-1 [39] with SV40 promoter-containing luciferase reporter constructs with hormone-responsive elements, including elements responsive to AR only (Rhox5 AR elements (AREs) and Eppin-AREs) or to both AR and Glucocorticoid Receptor (GR) (Tat-GRE, a known binding element for both AR and GR [40]) (**Fig 5D** and **S9A Fig**). Stimulation of MSC-1 cells transfected with the Rhox5-ARE reporter with the androgen 5α-dihydrotestosterone (5α-DHT), which activates the endogenous AR, induced a significant increase in luciferase activity (**S9B Fig**), with the concomitant transfection of AR strongly enhancing Rhox5-ARE reporter activity (**S9C Fig**). Importantly, pre-treatment of cells overexpressing AR with the p110β inhibitor TGX-221 decreased 5α-DHT-induced luciferase expression driven by Rhox5-AREs (**Fig 5E**), Eppin-AREs (**S9D Fig**) and Tat-GREs (**S9E Fig**). While the observed decrease in AR-dependent transcriptional activation upon p110β inhibition was modest in cell culture, a strong impact on the expression of Rhox5 was seen *in vivo*, with a 29%, 59% and 81% decrease in gene expression in adult SCβ-DEL, p110β^D931A/WT^ and p110β^D931A/D931A^ males, respectively, compared to WT mice (**Fig 5F and 5A, right panel**). Taken together, these data show that p110β activity regulates AR transcriptional activity, contributing to the expression of the SC-specific AR target *Rhox5 in vivo*.

### The other ubiquitous class I isoform p110α also regulates fertility

The testis phenotype upon global p110β inactivation is stronger than that observed in SCARKO mice (see above). This is possibly due to an additional role that p110β has directly in the spermatogenic germ cell lineage, in addition to its ability to regulate AR signalling in SCs, for example its previously reported involvement in c-kit receptor-positive male germ cells [12].

**Fig 6:**
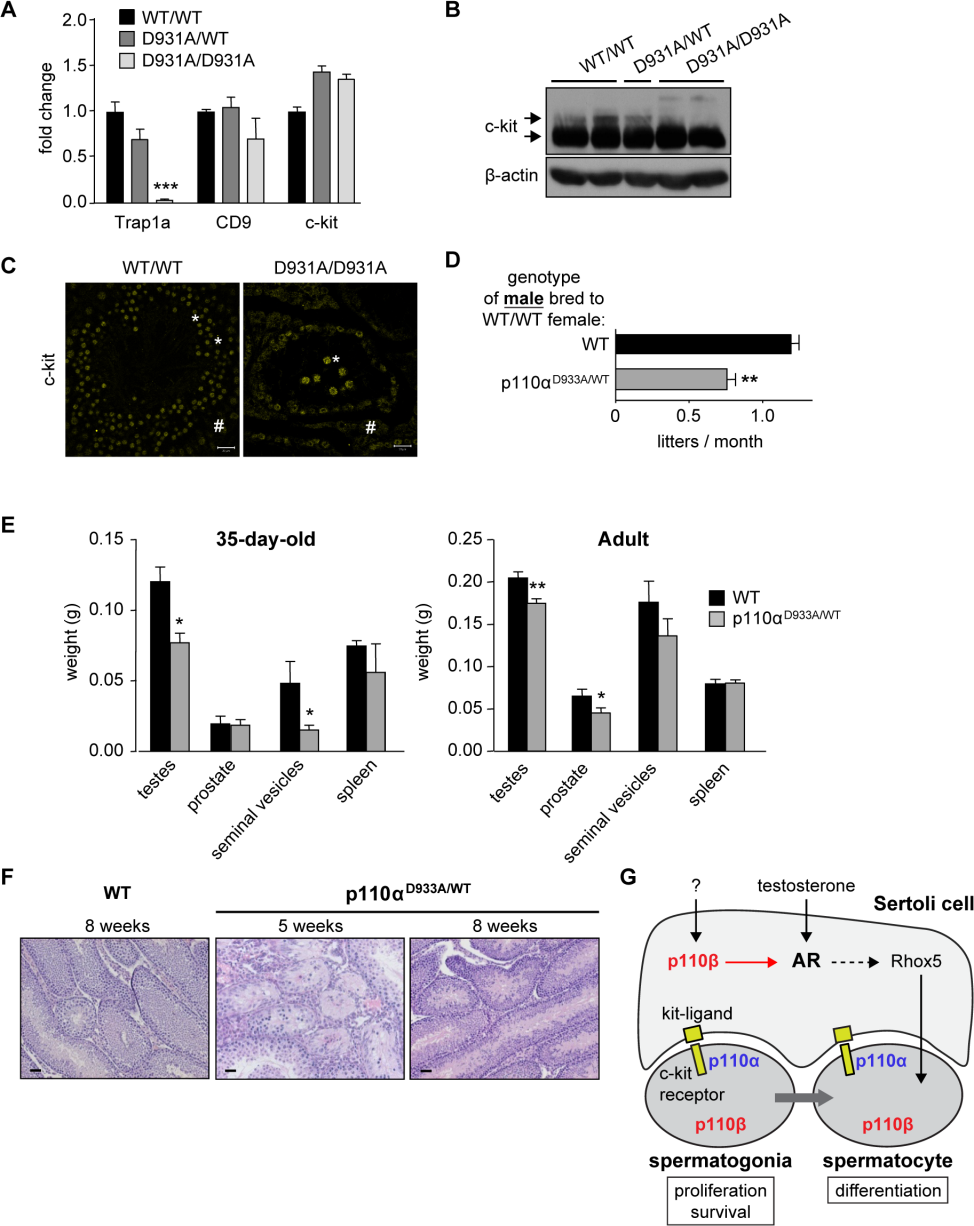
p110α couples to c-kit in testes and regulates male fertility. **A**) mRNA expression of stem cell markers in testes of adult mice of the indicated genotypes as determined by RT-qPCR and normalized with 18S expression and corrected for total testis weight (n = 5). Student's t-test: ***, p<0.001. **B**) Western blot analysis of c-kit expression in adult testes of mice with the indicated genotypes (n = 4). Arrows point to the 100 kDa and 150 kDa forms of c-kit. **C**) Immunofluorescence using anti-c-kit TRITC-labeled antibodies in adult WT and p110β^D931A/D931A^ testes (n = 3). Scale: 20 μm. * c-kit-positive germ cells; # c-kit-positive Leydig cells. **D**) Breeding efficiency of p110α^D933A/WT^ males crossed with 2 WT females over a 4 month period (C57BL/6 background). Unpaired t-test: **, p<0.01. **E**) Weight of reproductive organs in P35 (n>3) and adult 12-week-old WT and p110α^D933A/WT^ mice (n>6). Mann-Whitney: *, p<0.05. **F**) H&E staining of testes of D35 and adult WT and p110α^D933A/WT^ mice (n>3). Scale: 20 μm. **G**) A schematic representation of the roles of p110α and p110β in the regulation of male fertility.

An important question is also whether other class IA PI3K isoforms than p110β are involved in the regulation of fertility. The male fertility phenotype of the p110β^D931A/D931A^ mice appears to be less pronounced compared to that of c-kit-p85 null mice (knockin mice in which c-kit can no longer interact with the p85 regulatory subunit of class IA PI3Ks; [19,20]). In the c-kit-p85 null mice, c-kit expression is drastically reduced in mutant seminiferous tubules already at P8 [19] and the spermatogenic germ cell pool is fully depleted at P21 [20]. In contrast, despite a strong reduction in the mRNA expression of the stem cell marker Trap1a (**Fig 6A**), the testes of p110β^D931A/D931A^ adult males still showed mRNA expression of the CD9 and c-kit stem cell markers (**Fig 6A**) and protein expression of c-kit (**Fig 6B**), demonstrating that they contained germ cells. In addition, some c-kit-positive spermatogonial cells, surrounded by c-kit-positive Leydig cells (respectively indicated by * and # in **Fig 6C**), were present in the seminiferous tubules of p110β^D931A/D931A^ males. These findings suggest that another class IA PI3K isoform than p110β could be involved in spermatogonial signalling, possibly downstream c-kit.

We therefore assessed the possible contribution of p110α to male fertility, using mice with a kinase-dead knockin allele of p110α [22,23]. Homozygous p110α^D933A/D933A^ mice are embryonic lethal [22] but heterozygous p110α^D933A/WT^ males were found to be subfertile (**Fig 6D**) with a significant decrease in testis size at 35 days after birth and in the adult stage (**Fig 6E**). An incompletely penetrant (2 mice out of 5) mixed atrophy of seminiferous tubules of p110α^D933A/WT^ was observed at 5 weeks of age. This atrophy was found to be reversed at 8 weeks of age (**Fig 6F**). In addition, p110α^D933A/WT^ females, when crossed with WT males, had a 35% reduced average litter frequency, compared to WT mice (**S10 Fig**). In contrast, analysis of homozygous p110δ kinase-dead testes showed no significant defects in ageing mice (**S11 Fig**).

Given that all class IA PI3K isoforms bind p85 [41], the association to the c-kit receptor of all p85-bound p110 isoforms is expected to be impaired in c-kit-p85 null mice. We found that in unstimulated 12-week-old testes, only p110α, but not p110β or δ, co-immunoprecipitated with c-kit (**S12 Fig**), whereas p110α and δ, but not p110β, co-immunoprecipitated with c-kit in spleen (**S12 Fig**), a tissue that is enriched in leukocytes and in which p110δ is known to transmit c-kit signalling [21,41,42]. These data suggest that only p110α contributes to c-kit signalling in adult testes, while p110β is not significantly recruited to c-kit receptor at this developmental stage.

These data show that both p110α and p110β, but not p110δ, contribute to male and female fertility in mice, with p110α, as a tyrosine kinase-linked class I PI3K, most likely executing this biological function through c-kit. The role of p110α in Sertoli cells is unknown.

## Discussion

Using a mouse model of constitutive inactivation of the ubiquitously expressed p110β isoform of PI3K, we document that only few mice with inactive p110β survive into adulthood, for reasons that are unclear at the moment. Interestingly, the only apparent phenotypes in the p110β kinase-dead mice that are born are subfertility in females and complete infertility in males. The importance of PI3K signalling in fertility was initially uncovered using mice in which the c-kit tyrosine kinase, an essential regulator of fertility in germ cells, was engineered to no longer interact with all class IA PI3Ks [19,20].

Our data reveal that both of the ubiquitously expressed class IA isoforms, p110α and p110β, regulate fertility in male germ cells (**Fig 6G**), with no fertility phenotypes observed upon full inactivation of p110δ, a leukocyte-restricted class I PI3K isoform.

A recent study demonstrated that the p110β isoform signals downstream of c-kit [12], uncovering a potential germ-cell intrinsic function of p110β in mice. However, the male fertility phenotype of p110β kinase-dead mice differs from that of c-kit/PI3K mutant mice, pointing to an additional, germ cell-extrinsic, function of p110β in the regulation of male fertility. Indeed, the testicular phenotype of mice with inactive p110β is reminiscent of that of mice with defective SCs, which are known to control the formation of the lumen of seminiferous tubules, attachment of the germinal cell lineage and efficient sperm formation and maturation [25,27,32]. Little is known about PI3K function in male germ cell support cells, with some evidence for a role of PI3K signalling in primary culture of SCs [43]. Importantly, SC-specific inactivation of p110β also led to male sterility, highlighting its important role in these support cells. Indeed, a decrease in the mRNA expression of the homeobox gene *Rhox5*, critical for the full efficiency of meiosis [29,32,34], was observed upon SC-specific genetic deletion of p110β as well as upon global genetic inactivation of p110β, suggesting that the catalytic activity of p110β was also important for SC function. Of note, the progenitor germ cell marker Trap1a was also found to be decreased in both mouse models (**Fig 4E** and **Fig 6A**), although the *in vivo* implication of this is currently unknown.

The male fertility phenotype of SC-selective p110β inactivation was less prominent than upon systemic p110β inactivation, in that it did not affect the germ cell composition of the mice, further suggesting potential germ cell-intrinsic roles for p110β, such as in c-kit-positive sperm cells. Of note, we cannot rule out that p110α also plays a role in SCs, as it is expressed in this cell type (**Fig 4A**). Taken together, our data show that the previously reported male infertility phenotype upon p110β inactivation [12] is not limited to a potential germ cell-intrinsic role of p110β in c-kit signalling, but is also related to an important role for p110β in the SC support cells.

The fertility phenotype of mice with inactive p110β strongly resembles that of mice with SC-selective deletion of the AR (SCARKO mice; [29]). We found that p110β activity regulates the expression of SC-specific genes that are essential for the differentiation of germ cell lineage and known to be regulated by AR [40]. Previous work has also implicated p110β as a positive regulator of AR transactivation in prostate cancer cell lines [44] and PI3K/mTOR signalling has been shown to either positively or negatively modulate AR transactivation both in prostate cancer cell lines and genetic mouse models of prostate cancer [45,46].

At present, the upstream signals that activate p110β in SCs are unknown. Between P10 and P15, the later time point being the one at which the p110β-linked phenotype becomes largely apparent in the testes, SCs regulate the induction of germ cell differentiation through the combined action of the AR and FSH, a ligand that signals through the FSH receptor (FSHR), a GPCR only expressed SCs [47,48]. FSHR deletion in mice mildly perturbs SC function and the progression of germ cells through spermatogenesis but when combined with AR deletion in SCs severely blocks this process [48]. As p110β mainly signals downstream of GPCRs [9–11,49,50], it is conceivable that p110β could mediate some of the action of FSH, and in particular the potential synergistic activity of FSH signalling on AR function. This remains to be investigated further.

We find that p110β activity modulates the transcriptional activity of the AR on DNA response elements from *Rhox5* or *Eppin* promoters. However, the exact way in which the lipid kinase activity of p110β signals to the AR is currently unclear. Although p110β is expected to act mainly in the cytosol, recent reports suggest that it could also act inside the nucleus where it has been found to regulate DNA repair and replication [51,52] and to directly interact with the AR in ChIP assays [44].

Female mice with full inactivation of p110β had a significant reduction in litter size and frequency. This might be explained by our finding that p110β activity (either in the maternal environment and/or intrinsically in the developing egg) contributes to the transition of explanted 2-cell embryos to the morula/blastocyst stage *in vitro*. These data are in line with previously published evidence, using PDK1 or PTEN inactivation in oocytes, which show that maternal PI3K signalling is crucial for embryonic genome activation and preimplantation embryogenesis in mice [53]. Preimplantation embryos may generate intrinsic signals that promote their survival and development, with paracrine/autocrine factors activating intracellular signalling events needed for early embryonic development [54,55]. Class I PI3K activity is known to contribute to the constitutive PtdIns(3,4,5)P_3_ lipid synthesis observed in mouse preimplantation embryos [56]. Moreover, granulosa cells surrounding the oocyte were shown to act via the PI3K/Akt/mTOR pathway to promote the translation of maternal oocyte mRNAs that are critical for preimplantation embryo development [57]. It is possible that p110β signalling downstream of the GPCR agonist LPA [9], known to be important in preimplantation embryos [58], contributes to the embryonic lethality upon p110β inactivation.

In cancer, p110β is often, but not always, the key PI3K isoform in cells with inactive PTEN [50,59–61]. p110β-selective drugs (such as GSK2636771 [62], clinicaltrials.gov identifier NCT01458067) are currently being tested in cancers with inactive PTEN, including prostate cancer. Our data suggest that such compounds, but also the broader spectrum class I PI3K inhibitors that hit both p110α and p110β, might have side-effects on human fertility.

Our data also provide a new lead for the development of male contraceptives. Indeed, in an organismal developmental context, p110β regulates AR targets only in SCs but not in other AR-responsive tissues, including prostate, seminal vesicles and epididymal/retroperitoneal fat, which would ensure minimal off-target effects of a p110β inhibitor. A male contraceptive should be free from side effects with a reversible action on sperm once the "male pill" is no longer taken. Our data suggest that p110β inhibitors could meet these requirements, with no overall phenotypes in p110β-deficient males other than sterility, due to a highly specific blockade of sperm maturation from spermatogonia to the primary spermatocyte stage (**Fig 3B**), while retaining most of the spermatogonial pool of cells that are at the origin of sperm development. Disorders of male and female fertility are on the increase. Our findings have additional potential clinical implications for unraveling mechanisms of idiopathic male and female infertility. Idiopathic non-obstructive azoo/oligozoospermia is a major health problem, accounting for about 30% of all male infertility cases. It is likely, and widely speculated, that novel mutations in genes regulating spermatogenesis will be discovered as causes of such situations. It is tempting to speculate that *Pik3cb* could be one of such candidate genes.

## Materials and Methods

### Reagents

Small molecule inhibitors were dissolved in DMSO, with final concentration of DMSO in the assays maximally 0.2%. TGX-221 was from Cayman. Some antibodies to class IA PI3Ks (p110α and p110β) were generated in-house (for details, see Ref. [9]), p110β antibodies for immunoblotting was from Santa Cruz Biotechnology (sc-602). Additional antibodies were from Upstate (p85-pan; 06–195), BD Biosciences (p110α; 94520–150); Alexis (p110γ; clone H1); Cell Signaling Technology (pS473-Akt, pT308-Akt, Akt, pS176-IKKα, p-S240/244-S6 or S6); Santa-Cruz Biotechnology (pT202-pY204-p44/42, c-kit (C19 and M14)) and Sigma (α-tubulin, β-actin). Cell culture reagents were from Invitrogen. Dihydroxytestosterone 5α-DHT was from Sigma.

### Ethics statement

Mice were kept in individually-ventilated cages. All procedures and animal care were conducted under the UK Licence PPL 70/7447, in accordance with the UK Animals (Scientific Procedures) Act 1986, with local ethics approval at University College London.

### Mice

Embryos or pups from timed pregnant mice were dissected at different time points, and those from E13.5 pregnant mice from mixed C57BL/6 x 129S2/Sv or C57BL/6 background were used to prepare MEFs as described [9]. Breeding efficiency was analysed in cages with 1 male and 2 females.

### Histology

Necropsy was performed after perfusion of mice with 4% formalin. Organs were fixed for 24 h in 4% PFA, washed twice in water and stored in 70% ethanol until embedding in paraffin. For analysis of testes, fixation was in Bouin’s solution (overnight) rather than PFA. H&E staining was performed on 2 μM sections. The diameter of the seminiferous cords/tubules was measured at 400× magnification using an ocular micrometer calibrated with a stage micrometer (Hamamatsu). Between 100 tubules that were either round or nearly round were chosen randomly and measured for each animal. For IHC, cryosections were stained with antibodies to JAM-A or JAM-C (kind gift from Sussan Nourshargh, Queen Mary University London) or anti-goat c-kit (Santa Cruz; 1/400).

### Cell lysis and immunoprecipitation

Mouse tissue and cultured cells were lysed in 1% w/v Triton X-100 in 50 mM Tris.HCl, pH 7.4, 150 mM NaCl, 1 mM EDTA, supplemented with protease and phosphatase inhibitor cocktails. Protein concentration was quantified by the BCA method for tissue or Bradford assay for cell lysates. IP of c-kit was performed after preclearing lysates with Sepharose protein A/G. Proteins were resolved on 8% SDS/PAGE gels and immunoblotted as described [9].

### Cell transfection and luciferase reporter assay

The murine MSC-1 SC line was transiently transfected with pcDNA3 plasmids with or without the DNA sequence encoding Flag-tagged human AR, together with a Firefly luciferase expression plasmid driven by AR- and/or GR-responsive elements, as shown in **S9 Fig**. Two controls were applied: 1) Firefly luciferase expression was normalised to expression of a co-transfected plasmid in which Renilla luciferase is driven by the SV40 promoter and 2) the luciferase values were normalised to values from wells transfected only with the plasmid in which Firefly luciferase is driven by the SV40 promoter, to account for non-specific induction of gene expression. Data are expressed as fold-increase of normalized Firefly/Renilla ratios. All transfections were performed in triplicate in 6-well plates. Indicated are the mean induction factors ± SEM after stimulation with 5–50 nM of 5α-DHT for 24 h. The DNA elements used and their nucleotide sequence were as follows: Rhox5-ARE-1 (5'-AGATCTCATTCTGTTCC-3'), Eppin-ARE (5'-AGAACTTGGTGTTCC-3) and TAT-GRE2 (5'-TGTACAGGATGTTCT-3') and were described in detail in [40,63].

### mRNA expression analysis

Tissue samples of (P10, P15 and P35 and week 8 and 12) testes were collected and snap-frozen in liquid nitrogen. All samples heterozygous for p110β were from mice on the C57BL/6 background. cDNA was synthesized from DNaseI-treated total RNA (RNeasy kit, Qiagen, Chatsworth, CA) using Superscript II RNaseH^–^ reverse transcriptase and random hexamer primers (Invitrogen). Primer pairs spanning an intron were designed by Applied Biosystems or previously published in [29] (for details see supplemental data). For quantification of gene expression, the ABI Prism 7700 sequence detector PCR detection system (Applied Biosystems) was used with a two-step RT-quantitative-PCR protocol. Gene expression was corrected for well-to-well loading variation by expressing data as a ratio to 18S rRNA. All samples and standard curves were run in triplicate. Data are analyzed using relative standard curves to allow comparison between all samples. Normalization of data to the total weights of the testes was performed to take into account the differential composition due to differential development of spermatogenesis. For Illumina array, testes from WT and heterozygous mutant P10 pups (on the C57BL/6 background) were harvested and snap-frozen. Purified mRNA was subjected to a quality check (Experion) and subjected to Illumina array analysis (Mouse Ref8v2 arrays). Five samples from each genotype in duplicate were subjected to the analysis. Quality Control and normalization were performed using BeadStudio (Illumina). Statistical analyses were performed using Bioconductor (www.bioconductor.org) packages within the open source R statistical environment (www.r-project.org). After filtering, the Limma package for differential expression analysis was used. Significant changes in gene expression were detected using a False Discovery Rate (FDR) < = 0.05. Data are represented as fold modification in log 2.

### SC-specific deletion of p110β

p110β^flox/flox^ mice (C57BL/6 background) were crossed with SC-specific Cre expressing mouse line AMH-Cre (C57BL/6 background) [28]. For detection of Cre-mediated excision of exons 21 and 22 of the p110β catalytic domain, mRNA was extracted from the testis and transcribed into cDNA and used as a template for a nested PCR to amplify exon 16–24 of *Pik3cb* using primers located in exon 16 (5’-CACTCCTGCTGTGTCCGTACA-3’) and 24 (5’-TCAGTGCTTCCTCCTCGCTCT-3’) followed by amplification of exons 19–23 using primers located in exon 19 (5’-TTGGACCTGCGGATGCTCCCCTAT-3’) or exon 23 (5’-CGCATCTTCACAGCACTGGCGGA-3’). The generation of a 204 bp (base pair) PCR fragment in testis samples from AMH-Cre^+^p110β^flox/flox^ (SCβ-DEL) mice indicated successful splicing of exon 20 onto exon 23, resulting in the generation of a mRNA encoding an internally truncated p110β protein [9].

### *In vitro* culture of 2-cell embryos

6- to 8-week-old female mice of the indicated genotypes on a C57BL/6 x 129 mixed background were superovulated by intraperitoneal injection of 7.5 IU pregnant mare's serum gonadotrophin (PMSG, Intervet) followed 48 h later by injection of 5 IU human chorionic gonadotrophin (hCG, Intervet). Female mice were mated with males of the indicated genotype (mixed background) at the time of hCG administration, and two-cell embryos were collected from the oviducts 1.5 days later (E1.5) in HEPES-buffered KSOM (Specialty Media) supplemented with amino acids. The numbers of 2-cell embryos recovered from WT and p110β^D931A/D931A^ females were similar, suggesting normal ovulation upon p110β inactivation. Embryos were cultured for 4 days in a 5% CO_2_ incubator in KSOM supplemented with amino acids. In order to reach high-density culture, embryos were placed into small drops of KSOM under mineral oil, at a density of one embryo per μl (typically, 15–20 embryos in 15–20 μl drops), as previously described [56]. After microscopic scoring of the stage of development, each embryo was digested for 2 h at 55°C in 5 μl of tail digestion buffer (100 mM NaCl, 10 mM Tris pH 8, 25 mM EDTA, 0.5% SDS) with proteinase K and pronase E (0.4 μg/ml); the reaction was stopped with 45 μl of TE (Tris.EDTA pH 8.0) and genotyping PCR performed on 2 μl of the reaction as described above. Embryos with failed genotyping (14% of all embryos cultured) were not taken into account: 6% were blastocysts or morulas and 8% were developmentally arrested embryos.

### Statistical analysis

*In vitro* and *in vivo* parameters were compared between two groups using the non-parametric Mann–Whitney *U*-test or unpaired t-test; quantifications and in vitro parameters using Student's t-test.

## Supporting Information

S1 Fig Generation of the p110β^D931A^ allele. Genomic DNA covering the last 4 exons of the coding region of the *Pik3cb* gene was isolated from a 129/Ola library. Mutations (shown in S1F Fig) were introduced using standard PCR-based mutagenesis technique, resulting in the conversion of the conserved DFG motif in the kinase domain to AFG. An IRES-*lacZ*-MC1-*neo* marker/selection cassette, flanked by *loxP* sites, was inserted after the STOP codon in the last exon (24) of the targeting vector. The linearized targeting construct was transfected into E14 Tg22a embryonic stem cells. G418-resistant clones with targeted integration of the vector were identified by Southern blot analysis of *NheI*- or *XbaI-*digested genomic DNA using probes (b79-40 and b81-53, respectively), homologous to genomic sequences external and flanking to the vector homology arms. Male chimeras generated from clones with the mutated p110β allele were bred with C57BL/6 females and germline transmission confirmed by Southern blot analysis of tail DNA using 5’ (b79-40) and 3’ (b81-53) flanking probes of *NheI*- (left) or *XbaI*- (right) digested genomic DNA. Fragment sizes revealed using the b79-40 probe on *NheI*-digested genomic DNA were 11.9 kb (for the endogenous locus) and 17.2 kb (for the targeted locus). Fragment sizes revealed using the b81-53 probe on *XbaI*-digested genomic DNA were 9.9 kb (for the endogenous locus) and 15.2 kb (for the targeted locus). The IRES-*LacZ*-MC1-*neo* selection cassette in p110β^D931A+cassette^ mice was removed by crossing these mice with a Cre deleter mouse line that expresses Cre recombinase in the germline [65]. A) Organization of the p110β (*Pik3cb*) gene locus. DFG is a conserved kinase domain motif in the C-terminal kinase domain of p110β. B) Linearised p110β targeting vector showing the mutation site AFG, the IRES-*LacZ*-MC1-*neo* marker/selection cassette and *lox*P sites. C) Targeted p110β allele. D) Targeted p110β allele following Cre recombinase-mediated deletion of the IRES-*LacZ*-MC1-*neo* marker/selection cassette. The b1-b2 PCR product is cleaved by *MscI* only if the mutation is inserted (not shown). (b1: 5’-GAGTCCTTGGATCAGGCGAATGG-3’; b2: 5’-TCTGACAGTAACTCCTCCCCACACC-3’). Keys: Exon sequences are represented by filled black rectangles, intron sequences by a black line. Restriction sites are *Nh = NheI*, *X = XbaI*, *M = MscI*, *C = ClaI*, *N = NotI*. Restriction sites are shown in italics. The relevant restriction fragments are highlighted by a horizontal line with double arrows. b79-40 and b81-53 probe fragments are represented by grey lines. The *lox*P sites are shown as pink triangles, with the pointed end indicating orientation. The positions of the primers used for PCR screening or verification of mutation are designated by red arrows or blue bars, respectively. E) Results of genotyping PCR after removal of the IRES-*LacZ*-MC1-*neo* marker/selection cassette, with size of PCR fragment of 455 bp for the WT allele and 605 bp for the D931A allele. F) Sequencing of genomic DNA isolated from WT or p110β^WT/D931A^ mice.
(EPS)

S2 Fig Impact of p110β^D931A^ mutation on PI3K activity and PI3K isoform expression. **A**) Lipid kinase activity in p110α or p110β IPs or class I PI3Ks isolated using immobilized phospho-tyrosine peptide, isolated from lung tissue (n = 3; mean ±SEM is represented; Student's t test: **, p<0.01). **B**) Western blot using the indicated antibodies in primary MEFs, lung and testis (n = 3; a representative experiment is shown). **C**) Lipid kinase activity in p110β IPs from lung tissue. The p110β inhibitor TGX-221 (100 nM) was added 15 min prior the *in vitro* kinase reaction (n = 3, the mean ±SEM is shown). Student's t test: *, p<0.05; **, p<0.01.
(EPS)

S3 Fig Impact of p110β inactivation on embryo survival and mouse growth. Intercrosses of heterozygous p110β^D931A/WT^ mice on a mixed C57BL/6 x 129S2/Sv background yielded a significantly lower fraction of homozygous p110β^D931A/D931A^ mice than expected based on a normal Mendelian distribution (≤13% from embryonic day (E) 10.5 onwards *versus* an expected ratio of 25%). Analysis of 278 embryos from p110β^D931A/WT^ intercrosses revealed an *in utero* lethality of p110β^D931A/D931A^ mice at two distinct time intervals: first between E8.5 and E10.5, followed by a second wave of lethality between E14.5 and E16.5 (A). On a C57BL/6 background, 1% of p110β^D931A/D931A^ mice survived until 4 weeks, compared to 7% on C57BL/6 x 129S2/Sv or 10% in C57BL/6 x 129S2/Sv x BALB/c mixed backgrounds. Before birth (from E16.5 onwards) and at weaning on day 28 postpartum, live homozygous p110β^D931A/D931A^ embryos and pups showed a decrease in size (B) and weight (C), respectively, compared to WT mice. At 12 and 33 weeks of age, however, WT and p110β^D931A/D931A^ mice had a similar body weight (D). **A**) p110β^D931A/WT^ mice were interbred, followed by genotyping of offspring at the indicated stages of development. Indicated is the percentage of homozygous p110β^D931A/D931A^ offspring yielded, with 25% being the expected percentage normal Mendelian distribution (dotted line). **B**) Size of dissected embryos (n>2) from a mixed C57BL/6 x 129 background. Mean ± SEM; Mann-Whitney; **, p<0.001. **C-D**) Weight of mice of the indicated age and genotype (n = 2–8). Mean ± SEM; Mann-Whitney; *p<0.05.
(EPS)

S4 Fig Impact of p110β inactivation on mouse organ weight. Average organ weights of 37-week-old males (n = 4) and 33-week-old females (n = 2). Mann-Whitney; **, p<0.001.
(EPS)

S5 Fig Impact of p110β inactivation on female fertility. A) Sections of ovaries of 12-week-old mice of the indicated genotypes (n = 5). Stages of follicle in ovary are indicated; CL indicates Corpus Luteus. Scale: 20 μm. B) Vaginal smears performed each day for 6 days on 12-week-old females of the indicated genotypes. Staining was with DiffQuick (n = 4). Representative pictures are shown. At oestrus, cornified non-nucleated, large, angular and irregular cells are observed (*). C) Number of 2-cell embryos after ovulation. Scale: 20 μm. D) Representative images of preimplantation embryos obtained after a 4-day culture of 2-cell embryos recovered from the oviducts of female mice of the indicated genotype. B: blastocyst; M: morula; 4/8: 4/8 cell stage. E) Validation of PCR efficiency in blastocysts. Three different blastocysts (B1, B2, B3) or a pool of 10 blastocysts (labeled as 10B) were digested for 2 h or overnight at 55°C, followed by a PCR for the mutated p110β allele using the indicated volume of the digestion mix. Tail digests were used as a positive control. Failed genotyping occurred at a 14% rate (6% were morulae or blastocysts; 8% were developmentally-arrested embryos).
(TIF)

S6 Fig CT-scan of testicular body area in WT and p110β^D931A/D931A^ mice. Testes (T), which have descended in the scrotum (arrow), are significantly reduced in size in p110β^D931A/D931A^ mice (12-week-old mice; n = 3). Representative mice are shown. Dotted lines surround testes.
(EPS)

S7 Fig Plasma levels of hormones in WT and p110β^D931A/D931A^ 12-week-old mice. (n≥4; mean ±SEM; Mann-Whitney, *, p<0.05)
(EPS)

S8 Fig Impact of p110β inactivation on testicular architecture. Immunolocalisation of cell-specific markers HSD3B (Leydig cells, labeled L), SOX9 (Sertoli cells), DDX4 (germ cells) and SMA (peritubular myoid cells) in WT testes compared to p110β^D931A/D931A^ testes at **A**) postnatal day 10 (P10), **B**) postnatal day 15 (P15), **C**) 13 weeks (13w) and **D**) 37 weeks (37w). Several tubules displayed cellular clusters (asterisk) within their lumen that were composed of Sertoli cells exhibiting elongated fibroblastic-like nuclei and the remnants of germ cells (scale bar: 100 μm). **E**) Absolute number (millions) of Leydig cells and Sertoli cells in the testes of 37-week-old WT and p110β^D931A/D931A^ mice (n = 3–4; mean ± SEM).
(TIF)

S9 Fig Transient transfection of luciferase reporter constructs containing AR-selective AREs or non-selective AREs in murine SCs. A) Schematic representation of the plasmids transiently transfected into MSC-1 cells. B-E) 24 h treatment with the testosterone analog 5α-DHT (50 nM) after transfection increases the transactivity of endogenous AR (B) or Flag-AR (C-E) on Rhox5 AREs (B,C), Eppin AREs (D) or TatGREs (E). TGX-221 (100 nM) was added together with 5α-DHT. Normalized Firefly/Renilla luciferase ratio is represented. Mean of 3 experiments done in triplicate ± SEM is shown, Students t-test, *, p<0.05.
(EPS)

S10 Fig Breeding efficiency of p110α^D933A/WT^ mice. Mice with the indicated genotypes were bred for a 6-month period (cages of 2 females with 1 male; > 3 couples) and the average number of litters per month was assessed (unpaired t-test: *, p<0.05; **, p<0.01).
(EPS)

S11 Fig H&E-stained sections of WT and p110δ^D910A/D910A^ testis. 1-year old mice on Balb/c or mixed C57/B6x SV129 backgrounds.
(TIF)

S12 Fig Recruitment of class IA PI3K isoforms to c-kit in 12-week-old spleen and testes. c-kit IPs and total lysates of the indicated tissues were run on SDS-PAGE and immunoblotted with the indicated antibodies. The arrow points to the location of the p110β protein; * indicates the non-specific band upon p110β blotting; arrowheads point to the 100 kDa and 150 kDa forms of c-kit.
(EPS)

S1 Text Detailed materials & methods. (DOCX)

S1 Table H&E-stained tissues subjected to histological analysis. (DOCX)

S2 Table List of the 42 genes with ≥2-fold change in expression between p110β^D931A/WT^ and WT P10 testes: ≥ 2-fold downregulated (18 genes; p value: 0.0052–0.00013); ≥2-fold up-regulated (24 genes; p value: 0.015–0.00013). (DOCX)

S3 Table Function of the 42 genes whose expression differs ≥2-fold between p110β^D931A/WT^ and WT P10 testes. (DOCX)

S4 Table Comparison of gene expression profiles in P10 testes of p110β^D931A/WT^ mice (this study) and SCARKO mice [38]. Summary of the genes downregulated (indicated in green with downward arrow) or upregulated (indicated in orange with upward arrow) in SCARKO mice as compared to control. In p110β^D931A/WT^ P10 testes, the expression of these genes was either not affected (=), downregulated (↓), upregulated (↑) or not detected (ND). The expression of 26 and 42 genes was modified >2-fold in SCARKO and p110β^D931A/WT^ testes, respectively. Expression of 21 genes was detected in both experiments. Of these 21 genes, the expression level of 9 genes (43%) was found modified in both p110β^D931A/WT^ and SCARKO P10 testes (5 downregulated; 4 upregulated).
(DOCX)
