# Supplementary figures and images for: Novel Role for p110β PI 3-Kinase in Male Fertility through Regulation of Androgen Receptor Activity in Sertoli Cells
Source: PLoS Genet. 2015 Jul 1;11(7):e1005304. doi: 10.1371/journal.pgen.1005304 (PMC4488938; doi:10.1371/journal.pgen.1005304)

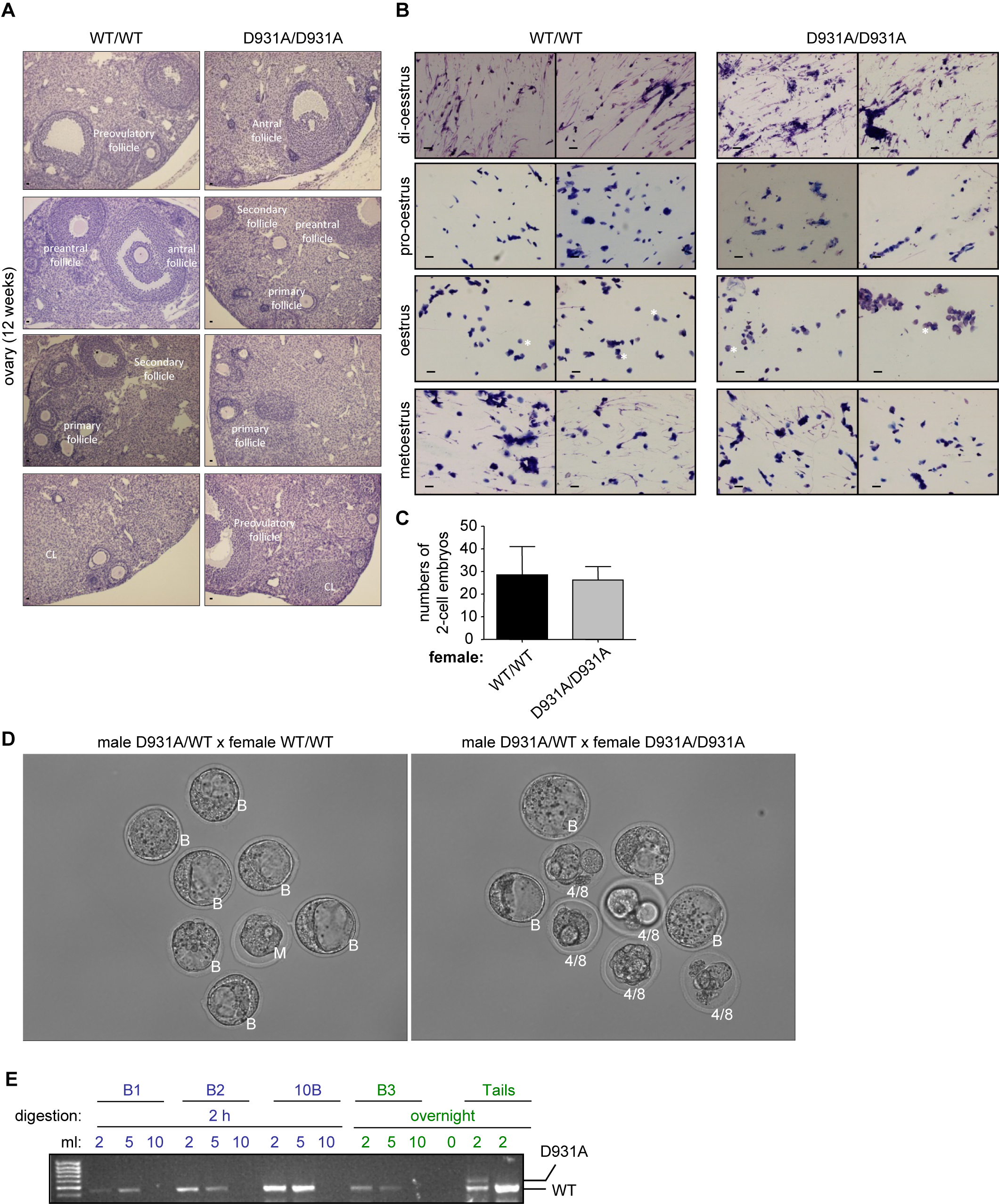

Supplement: S5 Fig — A) Sections of ovaries of 12-week-old mice of the indicated genotypes (n = 5). Stages of follicle in ovary are indicated; CL indicates Corpus Luteus. Scale: 20 μm. B) Vaginal smears performed each day for 6 days on 12-week-old females of the indicated genotypes. Staining was with DiffQuick (n = 4). Representative pictures are shown. At oestrus, cornified non-nucleated, large, angular and irregular cells are observed (*). C) Number of 2-cell embryos after ovulation. Scale: 20 μm. D) Representative images of preimplantation embryos obtained after a 4-day culture of 2-cell embryos recovered from the oviducts of female mice of the indicated genotype. B: blastocyst; M: morula; 4/8: 4/8 cell stage. E) Validation of PCR efficiency in blastocysts. Three different blastocysts (B1, B2, B3) or a pool of 10 blastocysts (labeled as 10B) were digested for 2 h or overnight at 55°C, followed by a PCR for the mutated p110β allele using the indicated volume of the digestion mix. Tail digests were used as a positive control. Failed genotyping occurred at a 14% rate (6% were morulae or blastocysts; 8% were developmentally-arrested embryos). (TIF) [file pgen.1005304.s005.tif]

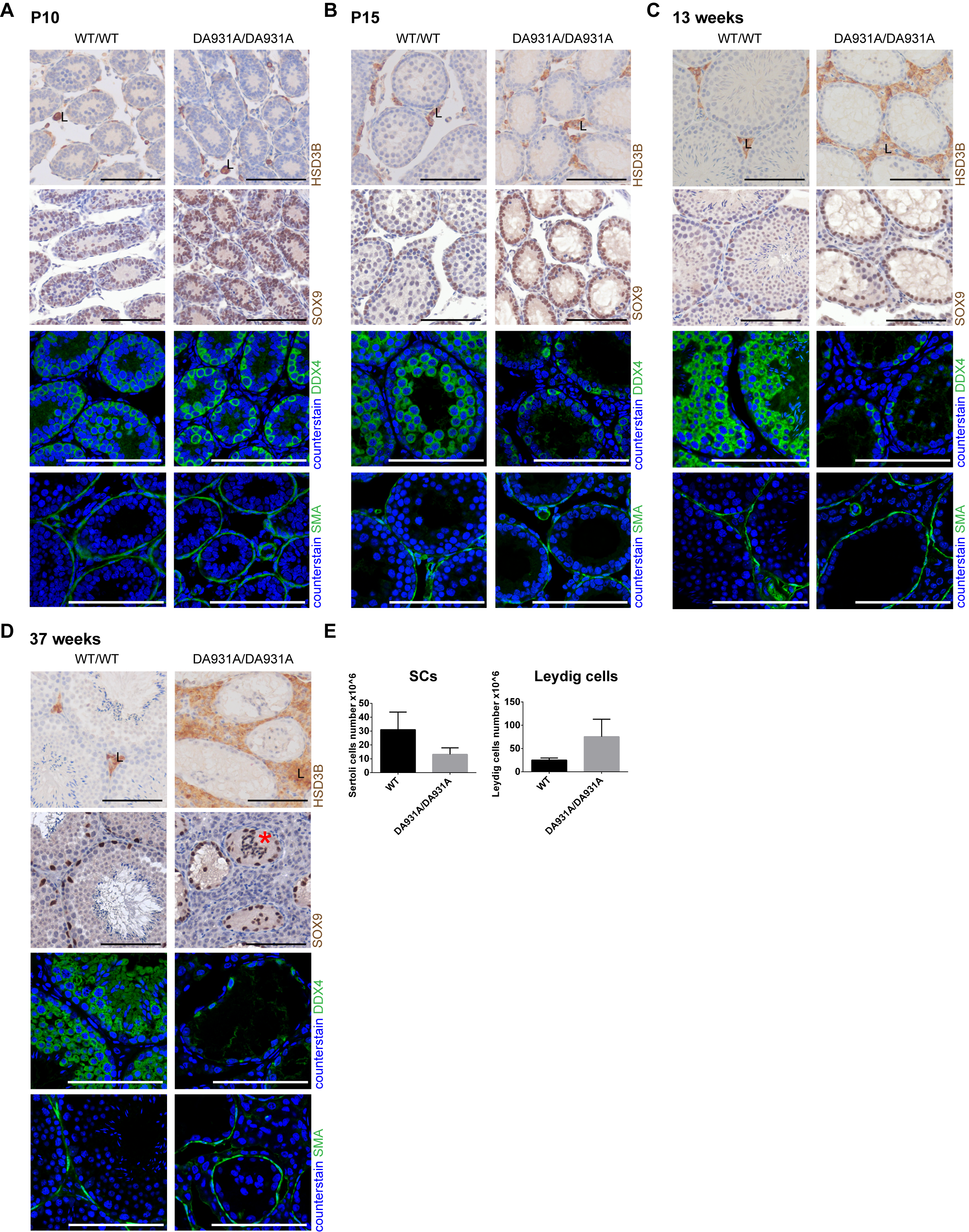

Supplement: S8 Fig — Immunolocalisation of cell-specific markers HSD3B (Leydig cells, labeled L), SOX9 (Sertoli cells), DDX4 (germ cells) and SMA (peritubular myoid cells) in WT testes compared to p110βD931A/D931A testes at A) postnatal day 10 (P10), B) postnatal day 15 (P15), C) 13 weeks (13w) and D) 37 weeks (37w). Several tubules displayed cellular clusters (asterisk) within their lumen that were composed of Sertoli cells exhibiting elongated fibroblastic-like nuclei and the remnants of germ cells (scale bar: 100 μm). E) Absolute number (millions) of Leydig cells and Sertoli cells in the testes of 37-week-old WT and p110βD931A/D931A mice (n = 3–4; mean ± SEM). (TIF) [file pgen.1005304.s008.tif]

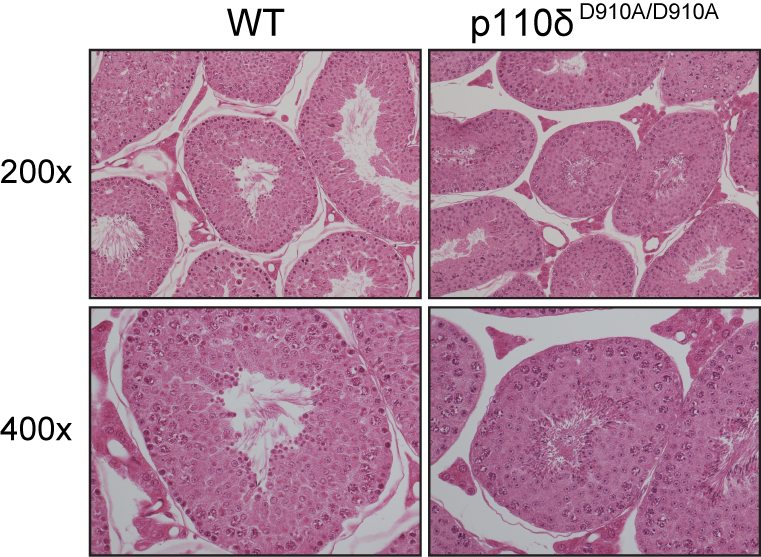

Supplement: S11 Fig — 1-year old mice on Balb/c or mixed C57/B6x SV129 backgrounds. (TIF) [file pgen.1005304.s011.tif]
