# Supplementary material for: Novel Role for p110β PI 3-Kinase in Male Fertility through Regulation of Androgen Receptor Activity in Sertoli Cells
Source: PLoS Genet. 2015 Jul 1;11(7):e1005304. doi: 10.1371/journal.pgen.1005304 (PMC4488938; doi:10.1371/journal.pgen.1005304)
Supplement: S1 Text — (DOCX) [file pgen.1005304.s013.docx]

### S1 Text

### Detailed Materials & Methods

### Mice

Mice with global inactivation p110α or p110γ or conditional inactivation of p110β were maintained in a C57BL/6 background (N>8). The IRES-*lacZ*-MC1-*neo* marker/selection cassette was removed by crossing to a Cre deleter mouse line [[65](#_ENREF_65)] with confirmation of germline recombination by a cross to WT C57BL/6 mice. Mice were backcrossed on selected mouse backgrounds for ≥5 generations before phenotypic characterisation. For mice on a mixed background, at least two intercrosses were performed before phenotypic characterisation. For fertility studies, all results with homozygous knockin mice were obtained using mice on both mixed C57BL/6 x 129S2/Sv and C57BL/6 background; data shown on heterozygous knockin mice are C57BL/6 only. Genotyping PCR conditions were as follows: [94°C for 3 min; (94°C for 30 sec; 60°C for 30 sec; 72°C for 30 sec) 39 times; 72°C for 7 min; 4°C] with primers b11 (5 µM), CTTAGGGAAGAGCGAGGA; Bseq2 (10 µM final), AAGAAGTATGTACACCTCTCT and NeoF2 (5 µM), CTGTCATCTCACCTTGCTCC. Sizes of the PCR product of WT and p110β^D931A+cassette^ alleles before removal of the selection/marker cassette were: WT: 455 bp; heterozygous mutant: 947 bp + 455 bp; homozygous mutant: 947 bp. After removal of the marker/selection cassette, the expected size of PCR products was as follows: WT: 455 bp; heterozygous D931A mutant: 455 bp + 605 bp [+ Heteroduplex, see [[22](#_ENREF_22)]]; homozygous D931A mutant: 605 bp. The genomic location of the primers is shown in **S1 Fig**. Sequences of additional probes/primers are the following: B79 AGGCCCAAGGATGATACAAC, B40 CCAAGCAGAGTGAGCATAT, B81 ACCCAGAGTGACAGCTGAGT, B53 CCTGACCAAGCATTTGCAAT.

**Hormone measurements**

Mice were anesthetized and exsanguinated by cardiac puncture, followed by plasma isolation and storage at -20°C until assayed. LH and FSH were measured by immunofluorometric assay (reagent from I. Huhtaniemi), testosterone was measured by ELISA (R&D).

**Oestrus determination**

Vaginal smears of 8- to 12-week-old females were obtained and smeared onto glass slides. The slides were stained with Giemsa stain (DiffQuick, Biostain Ready Reagent), and the different phase of the oestrus cycle; di-oestrus, pro-oestrus, oestrus and metoestrus were determined. The smears were scored by two independent observers.

### Cell culture and stimulation

Cells were cultured at 37°C in a humidified 5% CO_2_ atmosphere. Murine MSC-1 SCs were cultured in DMEM supplemented with 10% FBS, 100 units/ml penicillin and 100 μg/ml streptomycin. For MSC-1 cells, cells are starved in DMEM without phenol, and stimulated in charcoal-stripped serum (CS-FBS) to prevent from exogenous steroids in presence of DHT (5-50 nM).

#### PI3K isoform expression and lipid kinase assay

PBS was perfused through the heart of terminally-anaesthetized mice, followed by harvesting and freezing of the tissues in liquid nitrogen. Three independent samples for each genotype were used for PI3K activity assays as follows. Protein (500 µg) of total tissue lysate was isolated using either a GGYpMDMSKDESVDYpVPML phospho-tyrosine peptide matrix [Yp is phosphotyrosine; this peptide is coupled to Actigel beads (Sterogene USA) and contains the two consensus SH2 domain binding sequences found in the PDGF receptor], or antibodies to p110α or p110β. In the latter case, IPs using a rabbit anti–mouse IgG were used as controls. Kinase assays were performed using phosphatidylinositol(4,5)bisphosphate (PtdIns(4,5)P_2_) (Lipid Products, Redhill, Surrey, UK) as a substrate in kinase buffer (20 mM Tris.HCl pH7.4, 100 mM NaCl, 0.5 mM EGTA, 10 mM MgCl_2_, and 100 µM ATP/2.5 µCi [^32^P]-γATP. Preincubation with isoform-specific inhibitors was performed 15 min before the assay. Lipids were extracted twice in choloroform/methanol and resolved on silica gel 60 TLC plates in a solvent system propanol-1:2M acetic acid 13:7). Radioactivity in the phosphatidylinositol(3,4,5)trisphosphate (PtdIns(3,4,5)P_3_) spot was measured using a Molecular PhosphorImager FX (Bio-Rad, Hercules, CA). The activity present in the control IgG IPs was subtracted from that found in p110 immunoprecipitates.

**Primers used for quantitative RT-PCR**

Reactions contained 1x Mastermix (Syber green ABI or Eva green Life technologies) and 200 nM forward primer, 200 nM reverse primer [[38](#_ENREF_38)] or 1x Taqman Mastermix primer (ABI) and 1.25 µl probe mix with each Taqman probes (FAM for gene of interest, VIC for 18S), 2 µl of a 1:10 dilution of the cDNA sample (starting material for reverse transcription is 2 µM of purified mRNA), and made to a total volume of 25 µl with sterile water.

| **SYBR green** |  |  |
| --- | --- | --- |
| **Targeted gene** | **forward primer** | **reverse primer** |
| Rhox5 | TCATCATTGATCCTATTCAGGGTATG | CTCTCCAGCCTGGAAGAAAGC |
| PABP | CCTTCATCAGCCCCTTGCT | TGGGATCCTCGCCTGGT |
| TP1 | GCAAGAACCGAGCTCCTCAC | GGACGCTCTTCCGGTATTTTC |
| protamine-1 | CAGCAAAAGCAGGAGCAGATG | GGCGACGGCAGCATCTTC |
| A-Myb | GGTCTTCATCAAAACTTCAACACAA | TGAACAGGAATGTAAAACTGATTCTG |
| claudin 11 | GCTCCAAGGGCCTGTGGGC | TGTCAACAGCAGCAAGATGGCG |
|  |  |  |
| **Taqman probes** |  |  |
| Hsd3b6 | ABI_ID: Mm00834440_m1 |  |
| Insl3 | ABI_ID: Mm01340353_m1 |  |
| AR | ABI_ID: Mm00442688_m1 |  |
| c-kit | ABI_ID: Mm00445221_m1 |  |
| Trap1a | ABI_ID: Mm01298174_m1 |  |
| 18S | Life technologies _4310893E |  |

**Primary SC culture**

SCs were isolated from testes of 5- or 19-day-old Wistar rats (Iffa-Credo, Lyon, France). Animals were treated following the current ethical guidelines of the European Community 86/609/CEE. Following purification as reported previously [[66](#_ENREF_66)], SCs were seeded at 50,000 cells/cm^2^ on CellBind plates (Corning Life Sciences, Wilkes Barre, PA) in DMEM (Sigma Aldrich, St-Louis, MO, USA) complemented with penicillin (100 IU/ml), streptomycin (100 µg/ml), glutamine (2 mM), retinol (50 ng/ml), vitamin E (200 ng/ml) and human transferrin (5 µg/ml), all purchased from Sigma Chemical Co (St-Louis, MO). On average, SC cultures were 90% pure, quantified as previously described [[66](#_ENREF_66)].

**Immunolocalisation**

Slides were antigen-retrieved in a pressure cooker with 0.01 M citrate buffer (pH 6.0). To quench endogenous peroxidases activity, slides were incubated in 0.3% hydrogen peroxide (v/v) in TBS for 30 min at room temperature (RT). The nonspecific activity was blocked using the appropriate normal blocking serum for 30 min at RT followed by incubation overnight at 4°C with the primary antibody SOX9 (Millipore, 2383973); HSD3B (Santa Cruz, sc30820) DDX4 (Abcam, GR149231-2) or αSMA (Sigma-Aldrich, 032M4822). After washing, slides were incubated for 30 min at RT with the appropriate secondary antibody conjugated to peroxidase. For DAB localisation, development was undertaken using diaminobenzidine (Immpact DAB; VectorLabs, Peterborough, UK). The reaction was stopped in water. Slides were then counterstained with haematoxylin, dehydrated and mounted with Pertex mounting medium (Cell Path, Hemel Hempstead, UK). Slides were analysed and photographed using a Provis microscope (Olympus Optical, London, UK) fitted with a DCS330 digital camera (Eastman Kodak, Rochester, NY). For immunofluorescence, sections were incubated with fluorescein Tyramide Signal Amplification system ('TSA™', Perkin Elmer) diluted 1/50 for 10 min at room temperature according to manufacturer’s instructions. Sections were then counterstained in Sytox Green (Molecular Probes, life technologies, Paisley, UK) for 10 min at RT and mounted in PermaFluor mounting medium (Thermo Scientific, UK). Slides were imaged using an LSM 710 confocal microscope and ZEN 2009 software (Carl Zeiss ltd, Hertfordshire, UK). To assure the specificity of the stained tissue, sections incubated with no primary antibody were used as negative controls.

**Measurement of Leydig and Sertoli cell volume per testis**

The absolute number of Leydig cells (HSD3B-positive cells) and Sertoli cells (SOX9-positive cells) was determined by standard stereology techniques using Zeiss Axio-Imager microscope (Carl Zeiss) connected to a Hitachi HVC20 camera (Hitachi Denshi Europe) and a Prior automatic stage (Prior Scientific Instruments Ltd.). As previously described [[67](#_ENREF_67),[68](#_ENREF_68)], testes were stained for HSD3B and SOX9 prior to counting. Using Image-Pro Plus v7.0 with Stereologer Analyzer Pro (Media Cybernetics) random fields (~40 fields counted per animals) were selected and subjected to point-counting allowing the determination of the volume of each cell type nuclei (Leydig cell and SC). Data were converted to volume per testis when multiplied by the testis weight. Leydig cell and SC numbers were then calculated using the mean value for the nuclear volume as described by [[69](#_ENREF_69)]. (n=3-4 animals; means±SEM).
