# Supplementary material for: Novel Role for p110β PI 3-Kinase in Male Fertility through Regulation of Androgen Receptor Activity in Sertoli Cells
Source: PLoS Genet. 2015 Jul 1;11(7):e1005304. doi: 10.1371/journal.pgen.1005304 (PMC4488938; doi:10.1371/journal.pgen.1005304)
Supplement: S1 Table — (DOCX) [file pgen.1005304.s014.docx]

**S1 Table H&E stained tissues subjected to histological analysis.**

| Brain |
| --- |
| Spinal cord |
| Sciatic nerve |
| Pituitary |
| Eye |
| Harderian gland |
| Adrenals |
| Liver/gallbladder |
| Kidneys |
| Lungs |
| Thyroids/Parathyroids |
| Oesophagus/Trachea |
| Stomach/Duodenum |
| Jejunum |
| Ileum |
| Caecum |
| Colon |
| Rectum |
| Mesenteric lymph node |
| Thymus |
| Pancreas |
| Urinary bladder |
| Spleen |
| Heart |
| Aorta |
| Salivary glands |
| Parotid |
| Mandibular lymph nodes |
| Uterus |
| Vagina |
| Cervix |
| Ovaries |
| Testes |
| Epididymides |
| Seminal vesicles |
| Prostate |
| Mammary gland/skin |
| Muscle |
| Tongue |
| Sternum |
| Femur/Tibial joint |
| Nose |
| Retroperitoneal fat |
| Brown fat |
| Epididymal fat |
