# Supplementary material for: Novel Role for p110β PI 3-Kinase in Male Fertility through Regulation of Androgen Receptor Activity in Sertoli Cells
Source: PLoS Genet. 2015 Jul 1;11(7):e1005304. doi: 10.1371/journal.pgen.1005304 (PMC4488938; doi:10.1371/journal.pgen.1005304)
Supplement: S2 Table — (DOCX) [file pgen.1005304.s015.docx]

**S2 Table List of the 42 genes with ≥2-fold change in expression between p110β^D931A/WT^ and WT P10** **testes**: ≥ 2-fold downregulated (18 genes; p value: 0.0052-0.00013); ≥2-fold up-regulated (24 genes; p value: 0.015-0.00013).

| Probe | Gene name | Log fold | Fold | p value |
| --- | --- | --- | --- | --- |
| downregulated |  |  |  |  |
| ILMN_2868131 | Pigt | -1,421348 | 0,241388 | 0,000127 |
| ILMN_2620406 | Nptx2 | -1,342733 | 0,261131 | 0,000231 |
| ILMN_3122845 | H1fx | -1,221120 | 0,294900 | 0,000762 |
| ILMN_2794608 | Bcat2 | -1,211089 | 0,297873 | 0,000306 |
| ILMN_2759669 | Scaf1 | -1,182681 | 0,306456 | 0,000560 |
| ILMN_1247646 | H1fx | -1,167296 | 0,311207 | 0,000494 |
| ILMN_3143246 | Ap2a1 | -1,165081 | 0,311897 | 0,000732 |
| ILMN_1250469 | Bcl9l | -1,143675 | 0,318646 | 0,001362 |
| ILMN_2981767 | Klc2 | -1,079304 | 0,339832 | 0,000201 |
| ILMN_2731578 | Espn | -1,070275 | 0,342914 | 0,000435 |
| ILMN_2764651 | Mt3 | -1,069807 | 0,343075 | 0,005245 |
| ILMN_2905866 | Cited2 | -1,067149 | 0,343988 | 0,000272 |
| ILMN_1222111 | Col15a1 | -1,061565 | 0,345914 | 0,000137 |
| ILMN_1222733 | Ptpn21 | -1,057601 | 0,347288 | 0,000201 |
| ILMN_2673566 | Eif4ebp2 | -1,036163 | 0,354814 | 0,001938 |
| ILMN_1252204 | Sepw1 | -1,035977 | 0,354880 | 0,002397 |
| ILMN_2602902 | Atp1b2 | -1,010467 | 0,364049 | 0,000494 |
| ILMN_2652187 | Cux1 | -1,003404 | 0,366629 | 0,001123 |
| upregulated |  |  |  |  |
| ILMN_1247543 | LOC100044692 | 1,016543 | 2,763624 | 0,000684 |
| ILMN_2764588 | Igfbp7 | 1,027477 | 2,794009 | 0,000508 |
| ILMN_2773540 | Thap4 | 1,032944 | 2,809323 | 0,000127 |
| ILMN_1221157 | Krt8 | 1,042561 | 2,836472 | 0,010057 |
| ILMN_2876755 | Defb42 | 1,042776 | 2,837082 | 0,015335 |
| ILMN_1231513 | Tacstd2 | 1,050731 | 2,859742 | 0,001165 |
| ILMN_2631771 | Cenpb | 1,059568 | 2,885125 | 0,000137 |
| ILMN_2722616 | Krt14 | 1,076635 | 2,934786 | 0,003341 |
| ILMN_2599861 | Defb11 | 1,088879 | 2,970941 | 0,008677 |
| ILMN_1254450 | Mdk | 1,096548 | 2,993812 | 0,000306 |
| ILMN_1255416 | Ly6a | 1,096830 | 2,994658 | 0,006965 |
| ILMN_2592718 | Cuzd1 | 1,105968 | 3,022149 | 0,020474 |
| ILMN_2773169 | Grb7 | 1,242149 | 3,463047 | 0,000127 |
| ILMN_1239050 | Defb20 | 1,242510 | 3,464298 | 0,004236 |
| ILMN_2960714 | Agpat4 | 1,259469 | 3,523550 | 0,014975 |
| ILMN_2910934 | Cd52 | 1,291679 | 3,638891 | 0,018265 |
| ILMN_2692412 | Defb2 | 1,368081 | 3,927807 | 0,002558 |
| ILMN_2712075 | Lcn2 | 1,432171 | 4,187782 | 0,025009 |
| ILMN_3141801 | Wfdc15b | 1,512085 | 4,536180 | 0,007440 |
| ILMN_2670038 | Cdh16 | 1,532893 | 4,631556 | 0,005298 |
| ILMN_2839313 | Actg2 | 1,591214 | 4,909706 | 0,000191 |
| ILMN_1220865 | Spink8 | 1,605071 | 4,978211 | 0,001741 |
| ILMN_2802487 | Defb29 | 1,627611 | 5,091696 | 0,023220 |
| ILMN_2738825 | Acta1 | 1,902541 | 6,702907 | 0,000968 |
