# Supplementary material for: Novel Role for p110β PI 3-Kinase in Male Fertility through Regulation of Androgen Receptor Activity in Sertoli Cells
Source: PLoS Genet. 2015 Jul 1;11(7):e1005304. doi: 10.1371/journal.pgen.1005304 (PMC4488938; doi:10.1371/journal.pgen.1005304)
Supplement: S3 Table — (DOCX) [file pgen.1005304.s016.docx]

**S3 Table Function of the 42 genes whose expression differs ≥2-fold between p110β^D931A/WT^ and WT P10 testes.**

|  | **> 2-fold downregulated** | **> 2-fold upregulated** |
| --- | --- | --- |
| translation | eIF4EBP2 (PI3K regulated) |  |
| signal transduction | PTPN21 (tyrosine phosphatase) | IGFBP7 |
|  | BCL9L (Wnt signalling) | GFRB7 |
|  | CUX1 (Wnt signalling) | Midkine (GF) |
| cytoskeleton/extracellular matrix/adhesion | collagenXV | keratin8 |
|  | MT3-MMP (metalloprotease) | keratin14 |
|  | Espin (tight junction) | acting2 |
|  | KLC2 (cell motility) | actina1 |
| endocytosis | Ap2a1 (component of clathrin-coated vesicles) |  |
| transcription factors | CITED |  |
|  | CUX1 |  |
| fertility | CITED2 | Actg2 |
|  | CUX1 | CUZD1 |
|  | Espin | Spink8 |
|  | KLC2 | defb42 (defensinb42) |
|  | Ap2a1 | defb11 (defensinb11) |
|  |  | defb29 (defensinb29) |
|  |  | Lcn2 (lipocalin2) |
|  |  | CD52 |
|  |  | Cenpb |
|  |  | Thap4 |
| stem cell markers |  | trop2 (tacstd2) |
|  |  | Ly6a |
| unknown or other functions | NPtx2 | LOC100044692 |
|  | Pigt | Defb20 (Defensinb20) |
|  | Bcat2 | Defb2 (Defensinb2) |
|  | Atp1b2 | Agpat4 |
|  | Scaf1 | Wfdc15b |
|  | Sepw1 |  |
|  | HistoneH1 |  |
