# Supplementary material for: Novel Role for p110β PI 3-Kinase in Male Fertility through Regulation of Androgen Receptor Activity in Sertoli Cells
Source: PLoS Genet. 2015 Jul 1;11(7):e1005304. doi: 10.1371/journal.pgen.1005304 (PMC4488938; doi:10.1371/journal.pgen.1005304)
Supplement: S4 Table — Summary of the genes downregulated (indicated in green with downward arrow) or upregulated (indicated in orange with upward arrow) in SCARKO mice as compared to control. In p110βD931A/WT P10 testes, the expression of these genes was either not affected (=), downregulated (↓), upregulated (↑) or not detected (ND). The expression of 26 and 42 genes was modified >2-fold in SCARKO and p110βD931A/WT testes, respectively. Expression of 21 genes was detected in both experiments. Of these 21 genes, the expression level of 9 genes (43%) was found modified in both p110βD931A/WT and SCARKO P10 testes (5 downregulated; 4 upregulated). (DOCX) [file pgen.1005304.s017.docx]

**S4 Table Comparison of gene expression profiles in P10 testes of p110β^D931A/WT^ mice (this study) and SCARKO mice [**[**38**](#_ENREF_38)**].** Summary of the genes downregulated (indicated in green with downward arrow) or upregulated (indicated in orange with upward arrow) in SCARKO mice as compared to control. In p110β^D931A/WT^ P10 testes, the expression of these genes was either not affected (=), downregulated (↓), upregulated (↑) or not detected (ND). The expression of 26 and 42 genes was modified >2-fold in SCARKO and p110β^D931A/WT^ testes, respectively. Expression of 21 genes was detected in both experiments. Of these 21 genes, the expression level of 9 genes (43%) was found modified in both p110β^D931A/WT^ and SCARKO P10 testes (5 downregulated; 4 upregulated).
